# Identification of a novel subgroup of endometrial cancer patients with loss of thyroid hormone receptor beta expression and improved survival

**DOI:** 10.1186/s12885-020-07325-y

**Published:** 2020-09-07

**Authors:** Daniel G. Piqué, John M. Greally, Jessica C. Mar

**Affiliations:** 1 Department of Systems and Computational Biology, Albert Einstein College of Medicine, 1300 Morris Park Avenue, Bronx, NY 10461 USA; 2 Department of Genetics, Albert Einstein College of Medicine, 1300 Morris Park Avenue, Bronx, NY 10461 USA; 3 Department of Epidemiology and Population Health, Albert Einstein College of Medicine, 1300 Morris Park Avenue, Bronx, NY 10461 USA; 4 Australian Institute for Bioengineering and Nanotechnology, The University of Queensland, Building 75, Cnr. College Rd & Cooper Rd, Brisbane, QLD 4072 Australia

**Keywords:** Endometrial cancer, Gene expression, Subgroup identification, Nuclear hormone receptors, Thyroid hormone receptor

## Abstract

**Background:**

Endometrial cancer (EC) is the most common gynecologic cancer in women, and the incidence of EC has increased by about 1% per year in the U. S over the last 10 years. Although 5-year survival rates for early-stage EC are around 80%, certain subtypes of EC that lose nuclear hormone receptor (NHR) expression are associated with poor survival rates. For example, estrogen receptor (ER)-negative EC typically harbors a worse prognosis compared to ER-positive EC. The molecular basis for the loss of NHR expression in endometrial tumors and its contribution to poor survival is largely unknown. Furthermore, there are no tools to systematically identify tumors that lose NHR mRNA expression relative to normal tissue. The development of such an approach could identify sets of NHR-based biomarkers for classifying patients into subgroups with poor survival outcomes.

**Methods:**

Here, a new computational method, termed *receptLoss*, was developed for identifying NHR expression loss in endometrial cancer relative to adjacent normal tissue. When applied to gene expression data from The Cancer Genome Atlas (TCGA), *receptLoss* identified 6 NHRs that were highly expressed in normal tissue and exhibited expression loss in a subset of endometrial tumors.

**Results:**

Three of the six identified NHRs – estrogen, progesterone, and androgen receptors – that are known to lose expression in ECs were correctly identified by *receptLoss*. Additionally, a novel association was found between thyroid hormone receptor beta (*THRB*) expression loss, increased expression of miRNA-146a, and increased rates of 5-year survival in the EC TCGA patient cohort. *THRB* expression loss occurs independently of estrogen and progesterone expression loss, suggesting the discovery of a distinct, clinically-relevant molecular subgroup.

**Conclusion:**

*ReceptLoss* is a novel, open-source software tool to systematically identify NHR expression loss in cancer. The application of *receptLoss* to endometrial cancer gene expression data identified *THRB*, a previously undescribed biomarker of survival in endometrial cancer. Applying *receptLoss* to expression data from additional cancer types could lead to the development of biomarkers of disease progression for patients with any other tumor type. *ReceptLoss* can be applied to expression data from additional cancer types with the goal of identifying biomarkers of differential survival.

## Background

Nuclear hormone receptors (NHRs) are a family of proteins encoded by 53 unique genes that generate changes in RNA transcription in response to external ligands [1]. The protein structure of NHRs consists of two domains – a ligand binding domain and a DNA-binding domain. Most NHRs have no known ligand and are not well characterized at a functional level. However, a small subset of NHRs – such as the estrogen (ER), progesterone (PR), androgen (AR), and thyroid receptors – and their ligands are well-studied because of their critical roles in reproductive physiology and development. For example, estrogen and progesterone mimetics are commonly used to regulate the uterine menstrual cycle as part of hormonal contraception regimens [2]. In addition, women who have severe hypothyroidism are more likely to have uterine menstrual disturbances [3, 4]. The NHRs thus play critical roles in both normal uterine physiology as well as uterine cancers.

Endometrial cancer arises from the inner lining of the uterus. The incidence of endometrial cancer in U.S. Caucasian women increased by 1–2% per year, on average, over the 10-year period from 2003 to 2012 [5, 6]. The loss of expression of NHRs, particularly of ER and PR, has been associated with poor clinical outcomes in endometrial carcinoma [7]. NHR expression may thus serve as a prognostic tool that can identify, at an early stage, subgroups of patients who are likely to develop an aggressive cancer in the future. ER and PR expression are also tightly correlated with the first of two classic histologic subtypes of endometrial cancer, type I and type II [8], which are used in combination with clinical features to risk-stratify patients and tailor treatment regimens. The identification of novel subgroups of endometrial cancer patients based on nuclear hormone receptor expression could aid in the development of new prognostic tools and therapeutic strategies to treat endometrial cancer.

Previous studies have highlighted the importance of nuclear receptor hormone expression in endometrial cancer. For example, unsupervised clustering of gene expression data from hundreds of patient tumors in The Cancer Genome Atlas (TCGA) uncovered a “hormonal” subtype of endometrial cancer associated with increased ER and PR expression and a favorable clinical prognosis [9]. The hormone receptor-positive subgroup was associated with phosphatase and tensin homolog (*PTEN*) mutations and a few tumor protein p53 (*TP53*) mutations and copy number alterations. These findings were consistent with previous associative studies that found links between the loss of ER or PR expression and poor clinical outcomes in endometrial cancer [10–12]. However, a targeted characterization of the role of the broader nuclear hormone receptor family in predicting clinical outcomes remains unaddressed. In part, this is because a computational framework for reliably detecting subgroups of patients who lose expression of NHRs relative to normal tissue has not yet been developed. The availability of a method for reliably subgrouping endometrial cancers patients based on their NHR status would facilitate the identification of novel biomarkers of disease progression.

Here, we develop a new computational approach termed *receptLoss* for identifying nuclear hormone receptors that lose expression in a subset of endometrial carcinomas relative to adjacent normal tissue [13]. Previously established associations between estrogen receptor (*ESR1*) and progesterone receptor (*PGR*) loss of expression and poor survival are confirmed by applying *receptLoss* to mRNA expression data from endometrial tumors in TCGA [14, 15]. In addition, a novel association between thyroid receptor beta (*THRB*) expression loss and improved 5-year survival in endometrial carcinoma is described. Finally, we show that *THRB* expression loss occurs independently of *ESR1* and *PGR* expression. In sum, these results describe a novel subgroup of endometrial cancers with differential survival based on *THRB* expression. In addition, *receptLoss* is a freely-available bioinformatics tool that can be utilized to identify subgroups of tumors that lose expression relative to normal tissue for any tumor type.

## Methods

### Data sources and sample selection

**Fig. 1:**
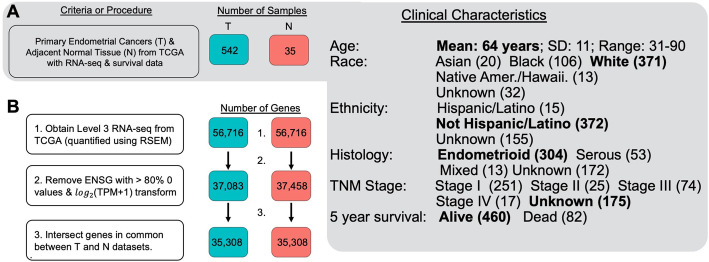
Transformation and filtering of RNA-sequencing data from endometrial carcinoma and adjacent normal samples. **a**. The numbers and clinical characteristics of endometrial carcinomas used in this study from the Cancer Genome Atlas (TCGA) are shown. **b** The filtering steps for the RNA-sequencing data are shown. Tumor and normal samples were filtered independently and then the intersection was taken to yield a common set of 35,308 genes that were used for subsequent analysis

**Fig. 2:**
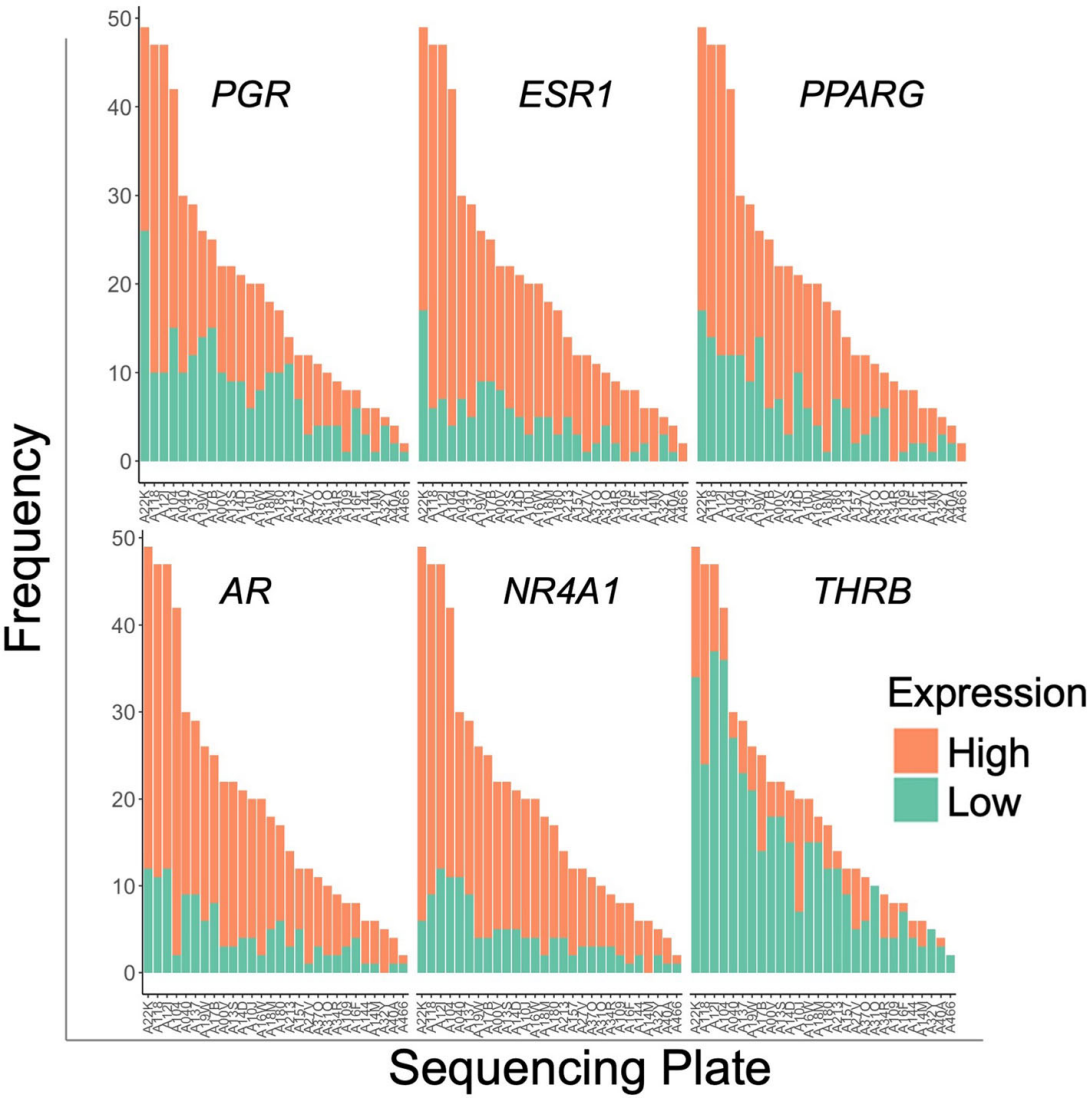
Binary expression values from NHRs are not significantly associated with sequencing plate. 29 sequencing plates, each of which sequenced between 2 and 49 samples, were tested for associations with NHR expression (either high or low) using a χ^2^ test (2 × 29 contingency table). Each of the 6 panels is a bar graph that shows the sequencing plate along the x-axis and the frequency along the y axis. The smallest Benjamini-Hochberg adjusted *q*-values were observed for THRB and PPARG (*q* = 0.10)

Level 3 FPKM mRNA and miRNA sequencing data from endometrial carcinoma and adjacent normal tissue along with clinical metadata were downloaded from the Genomic Data Commons (GDC) web server in January 2018 using the GenomicDataCommons and TCGAbiolinks R packages (Fig. 1) [16]. All samples from the TCGA endometrial adenocarcinoma cohort were selected [9, 16]. mRNA and miRNA-sequencing data were normalized and processed according to standard GDC protocols [17]. The mRNA FPKM output mapped to 56,716 ensembl (ENSG) gene ids and was converted to transcripts per million (TPM) and subsequently log_2_(TPM + 1) transformed to shrink the numeric range of the data. The miRNA FPKM data were similarly log_2_(TPM + 1) transformed. Following an initial filtering step (see below), mRNA-sequencing data were checked for confounders using principal component analysis. To test whether NHR expression values were associated with the sequencing plate, a technical variable, a Fisher’s exact test was performed (Fig. 2).

### Identification of NHRs with loss of expression in a subset of endometrial cancers

**Fig. 3:**
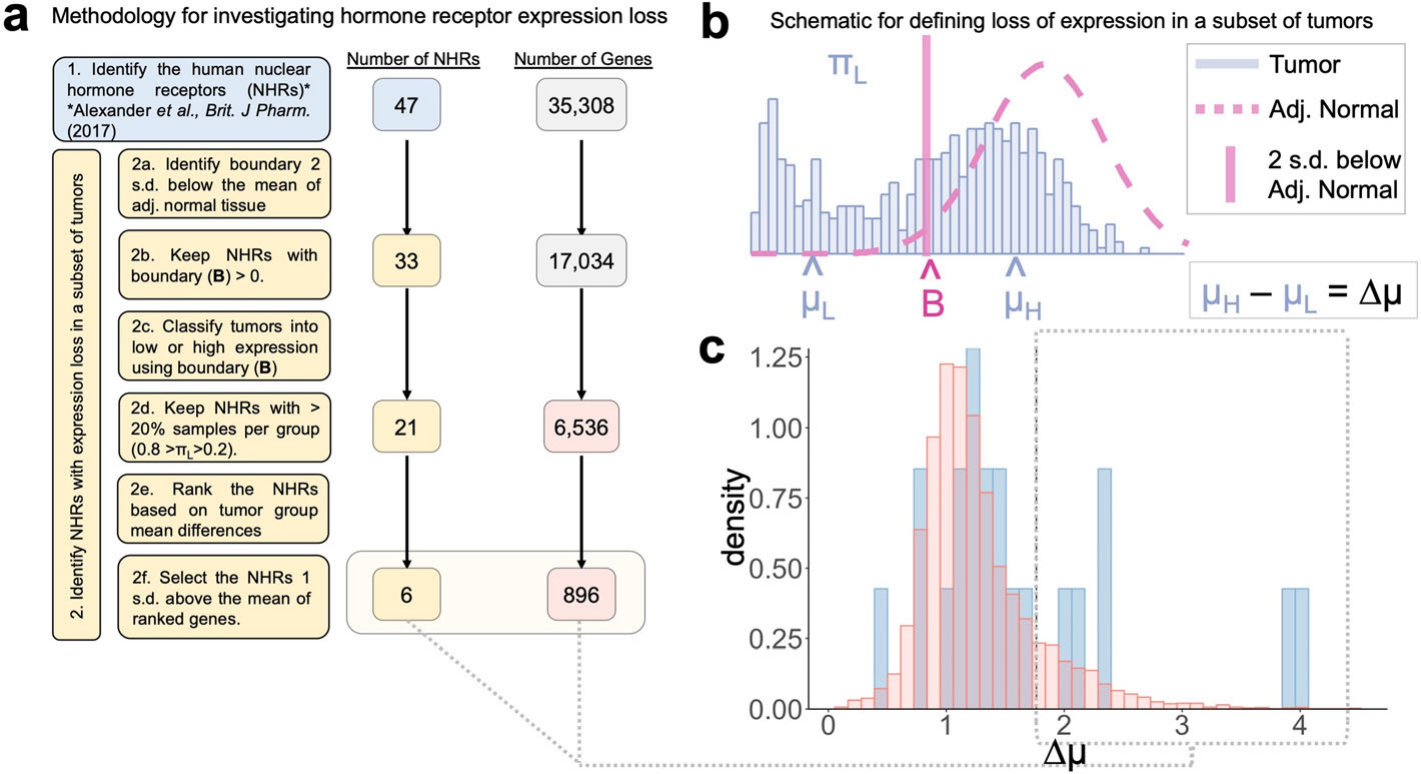
Overview of the approach for defining NHR expression loss in a subset of tumors. **a** Of the 53 NHRs with unique ENSG identifiers [1], 47 were present among the pool of 35,308 expressed genes. Filtering steps were performed in parallel for both nuclear hormone receptors and all other genes (see Methods). **b** Histogram of mRNA expression data for PGR (Progesterone receptor) in 542 endometrial tumors derived from TCGA (blue). Expression of PGR in 35 adjacent normal endometrial tissues is represented by the dotted line. The boundary (B) is the point 2 standard deviations below the mean of the normal tissue and defines the classification boundary between low and high expression in tumors. μ_L_ represents the mean of the group below the boundary, and μ_H_ reflects the mean of the group of tumors above the boundary. **c** Distribution of the ∆μ statistic across 6536 genes (peach) and across 21 NHRs (blue)

Filtering of mRNA transcripts was performed to narrow down the possible space of candidate NHRs (see Figs. 1 and 3a). To remove genes that were rarely expressed, genes with zero expression in either tumor or adjacent normal endometrial tissue across > 80% of individuals were excluded. Next, for each gene, a boundary was determined to separate tumors into low and high expression groups. Specifically, a boundary two standard deviations below the mean of expression levels in adjacent normal tissue was utilized. Genes with a negative boundary were removed. Tumors were then classified according to their expression above and below the adjacent normal tissue boundary (either “high” or “low”, respectively). Genes with less than 20% or greater than 80% of samples in either the high or low expression tumor group were excluded to allow for sufficient samples for downstream statistical analysis.

### Survival analysis

Survival analysis was performed using R using the log-rank test as implemented in the ‘survival’ R package [18]. *P*-values were adjusted for multiple testing using the Benjamini-Hochberg method.

### Mutation data sources and processing

All Tier 1 oncogenes and tumor suppressors were downloaded from the COSMIC database in December 2017 [19]. Mutation data were downloaded from the GDC, and mutations were called using the MuTect2 pipeline [20]. DNA mutations designated as “high-impact,” meaning that they likely impacted the protein structure or the splicing of an mRNA, were selected. A binary matrix was created from the tumor mutation data, where a 1 corresponded to whether the patient had 1 or more high-impact mutations in that gene, and a 0 indicated the absence of a mutation in that gene for that patient (Fig. 6a). Association studies between mutation and expression status were performed using a 2 × 2 Fisher’s exact test (see Fig. 6a) and corrected for multiple testing using the Benjamini-Hochberg method [21].

### Code availability

All analyses and plots were performed in the statistical language R (version 3.6.0). An HTML document created using knitR and RMarkdown contains the code and workflow for all analysis performed in this study (https://github.com/dpique/endometrial-paper/blob/master/2020_endomet_smry.html). An R package *receptLoss* is available on Github (https://github.com/dpique/receptLoss) that facilitates the identification of tumors with expression loss for any dataset with gene expression data from tumor and adjacent normal tissue. This package is also available from Bioconductor (https://bioconductor.org/packages/release/bioc/html/receptLoss.html).

## Results

### Leveraging expression heterogeneity to identify genes that lose expression in a subset of tumors

Our first objective was to develop an approach to identify nuclear hormone receptors (NHRs) whose expression is lost in a subset of endometrial tumors relative to adjacent normal endometrial tissue within a patient cohort. To accommodate low numbers of adjacent normal samples (*N* = 35) relative to tumor samples (*N* = 542), we assumed the adjacent normal data were generated from a single Gaussian distribution for each NHR. Then, using a lower bound defined by two standard deviations below the mean of this Gaussian distribution for the adjacent normal expression data (labeled as “B” in Fig. 3b), tumors were classified into two separate groups based on their expression values relative to this boundary. The advantage of this approach is that no assumptions are made as to whether the tumor data follow a particular distribution [22]. Therefore, patient subgroupings may be inferred in an unsupervised, distribution-independent manner from a low number of adjacent normal samples.

We applied the *receptLoss* approach to detect tumor-specific expression loss for the expression profiles of the 47 previously-identified NHRs that were captured by the filtered gene expression dataset [1]. Any NHRs that lost expression in a subset of endometrial tumors relative to normal tissue were selected after initial filtering (Fig. 3a, step 2b). An example of the distribution of one NHR in both tumor (blue histogram) and adjacent normal samples is shown in Fig. 3b. There are two subgroups of tumors, separated by a boundary (labeled as “B” in Fig. 3b) drawn by the threshold defined by two standard deviations below the mean of the adjacent normal tissue expression data. To quantify the degree of separation between the two tumor subgroups defined by the NHR expression in adjacent normal tissue, a ∆μ statistic was developed. The ∆μ statistic measures the difference between the means of the two tumor groups for each gene (Fig. 3c). The distribution of all ∆μ values for each of the 21 NHRs that met initial filters (see Methods and Fig. 3c, blue histogram) versus the 6536 genes in the genome is shown in Fig. 3c.

**Fig. 4:**
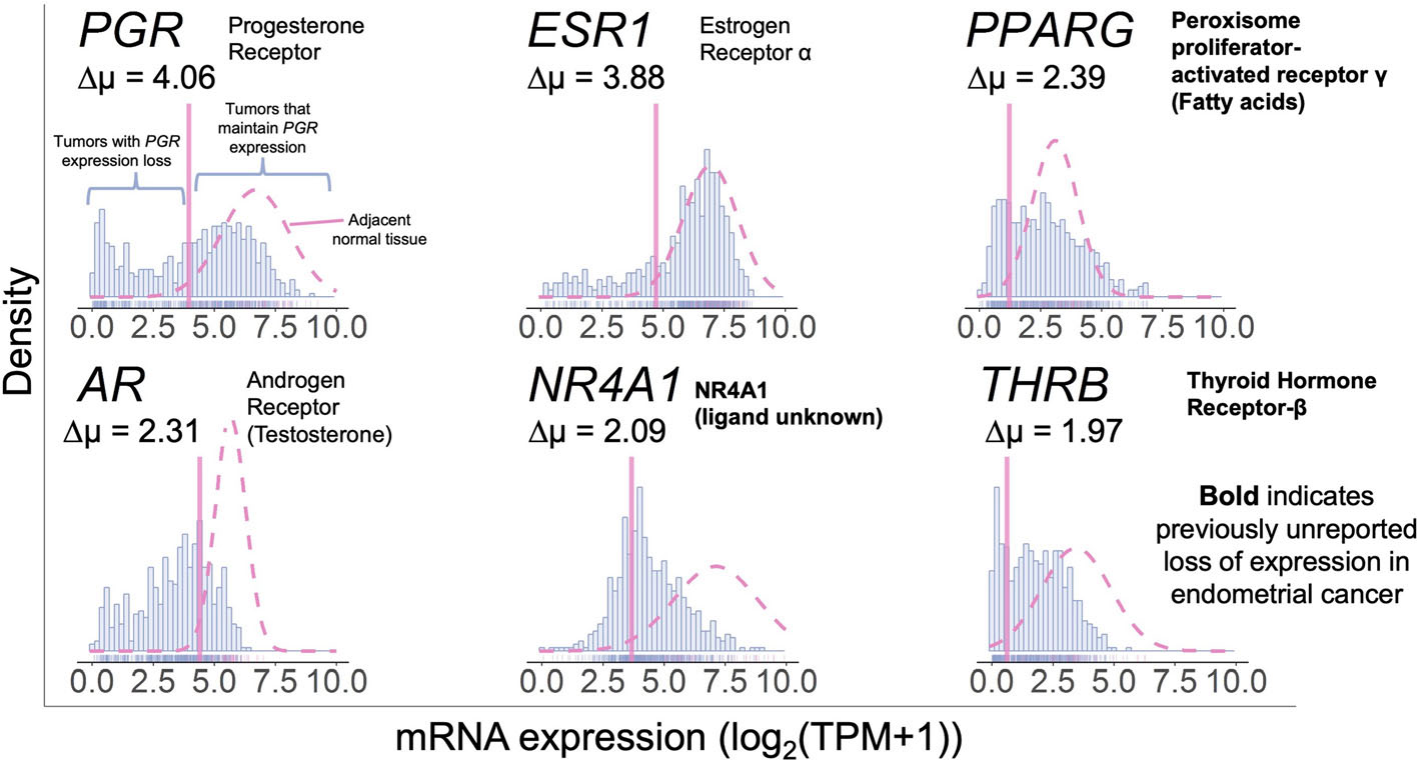
Identification of novel subgroups of endometrial cancers based on NHR expression levels relative to adjacent normal tissue. Genes are ordered by descending ∆μ statistic. Tumor data (*N* = 542) are represented by a blue histogram, and adjacent normal data (*N* = 35) are represented by a Gaussian distribution (pink dotted curve). A vertical pink line demarcates the lower boundary (two standard deviations below the mean) of the adjacent normal data. The ligands for the receptors are listed in parentheses, if not listed in the receptor name itself

Next, NHRs with ∆μ values that fell 1 standard deviation above all genes were selected. Six NHRs with relatively large ∆μ values exhibit a loss of expression in a subset of tumors relative to normal tissue (Fig. 4). Three of these NHRs – progesterone receptor (*PGR*), estrogen receptor 1 (*ESR1*), and androgen receptor (*AR*) – have been previously reported to lose expression in subsets of endometrial carcinomas [10, 23], which confirms that our approach can capture NHRs known to lose expression in endometrial carcinoma. The proportion of tumors that lose expression for each gene varies widely, along with the shapes of the gene expression distribution. These properties further illustrate how this approach is more flexible than standard analyses that assume identical distributions in both tumor and normal groups. The degree of expression loss is most prominent with *PGR*, which has the largest ∆μ value and whose tumor gene expression values are bimodally distributed. These results suggest that our approach adequately captures a distinct subset of NHRs that exhibit expression loss in a subset of tumors relative to normal tissue.

### The loss of *THRB* expression is associated with improved 5-year survival

**Fig. 5:**
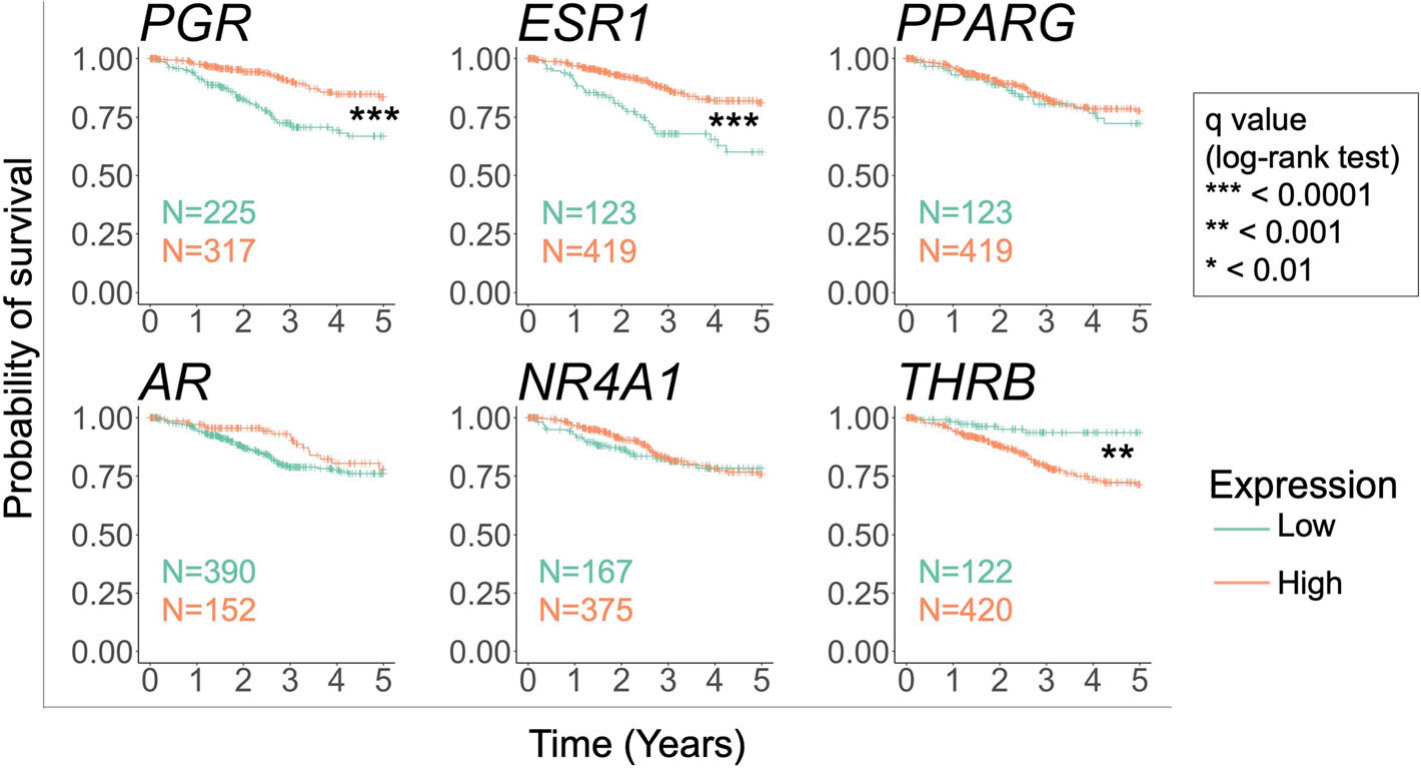
The loss of expression of THRB, ESR1, and PGR is associated with differences in 5-year survival. The number of samples within the low and high expression groups is shown within each Kaplan-Meier survival plot. Statistical significance was assessed using the log-rank statistic, and *P*-values were adjusted for multiple testing using the Benjamini-Hochberg method to obtain *q*-values. Asterisks indicate q values at the following thresholds: * = *q* < 0.01, ** = *q* < 0.001, *** = *q* < 0.0001

It has been observed that the loss of expression of estrogen and progesterone, two of the best characterized NHRs, is associated with worse outcomes in endometrial cancer [15]. The identification of new NHRs whose expression is associated with clinical outcomes could aid in the development of new prognostic biomarkers or therapeutic targets. Three out of the six NHRs (*PGR*, *ESR1*, and *THRB*) showed significant differences in five-year survival analysis between tumors that lost expression versus those that did not (log-rank test, *q*-value < 0.001, see Methods) (Fig. 5). Of note, the loss of *THRB* expression was associated with improved 5-year survival. This contrasts with the pattern observed for *PGR* and *ESR1*, wherein the loss of these receptors is associated with worse 5-year survival (Fig. 5). In the TCGA cohort, there were no significant differences between *AR* loss of expression and 5-year survival, though other studies have found conflicting results regarding AR expression and clinical outcomes in endometrial cancer [23, 24]. These results show a previously unreported relationship between *THRB* expression and endometrial cancer with regards to survival outcome.

### The loss of *THRB* expression is associated with TCGA molecular subtypes but not with traditional clinical prognostic factors

In order to identify additional clinical correlations with *THRB* expression loss, the relationship between *THRB* expression (loss versus no loss) and several clinical prognostic factors (stage, grade, histology, and TCGA molecular subtypes) was examined [9]. The clinical stage (χ^2^ test, *q* = 0.063), grade (χ^2^ test, *q* = 0.036), and histology (χ^2^ test, *q* = 0.031) of endometrial tumors were not significantly associated with *THRB* expression at the q < 0.01 level. However, there was a strong association (χ^2^ test, *q* = 4.94 × 10^− 4^) between the loss of *THRB* expression and the integrative molecular subtype defined by TCGA [9]. Among the 4 integrative molecular subtypes defined by TCGA, the microsatellite instability (MSI) subtype was significantly enriched among tumors that lose *THRB* expression (28/122 = 23.0%) compared with tumors that do not lose *THRB* expression (37/420 = 8.8%) (Fisher’s exact test, odds ratio = 3.07, *q* = 9.76 × 10^− 5^). Next, the relationship between *THRB* expression and the degree of MSI was examined using the MSI-specific classification approach reported by TCGA (χ^2^ test, *q* = 4.44 × 10^− 6^) [9]. Endometrial tumors that lost *THRB* expression had a greater proportion of tumors classified as high-grade MSI (MSI-high: 52/122 = 42.6%) relative to endometrial tumors that did not lose *THRB* expression (MSI-high: 75/420 = 17.9%) (Fisher’s exact test, odds ratio = 3.40, *q* = 1.12 × 10^− 7^). The previously unreported association between loss of *THRB* expression and microsatellite instability in endometrial carcinoma forms the basis for future mechanistic studies and links *THRB* expression loss to a molecular subtype established by TCGA.

### Loss of *THRB* expression is associated with high-impact mutations in *RNF43* and *NSD1*

**Fig. 6:**
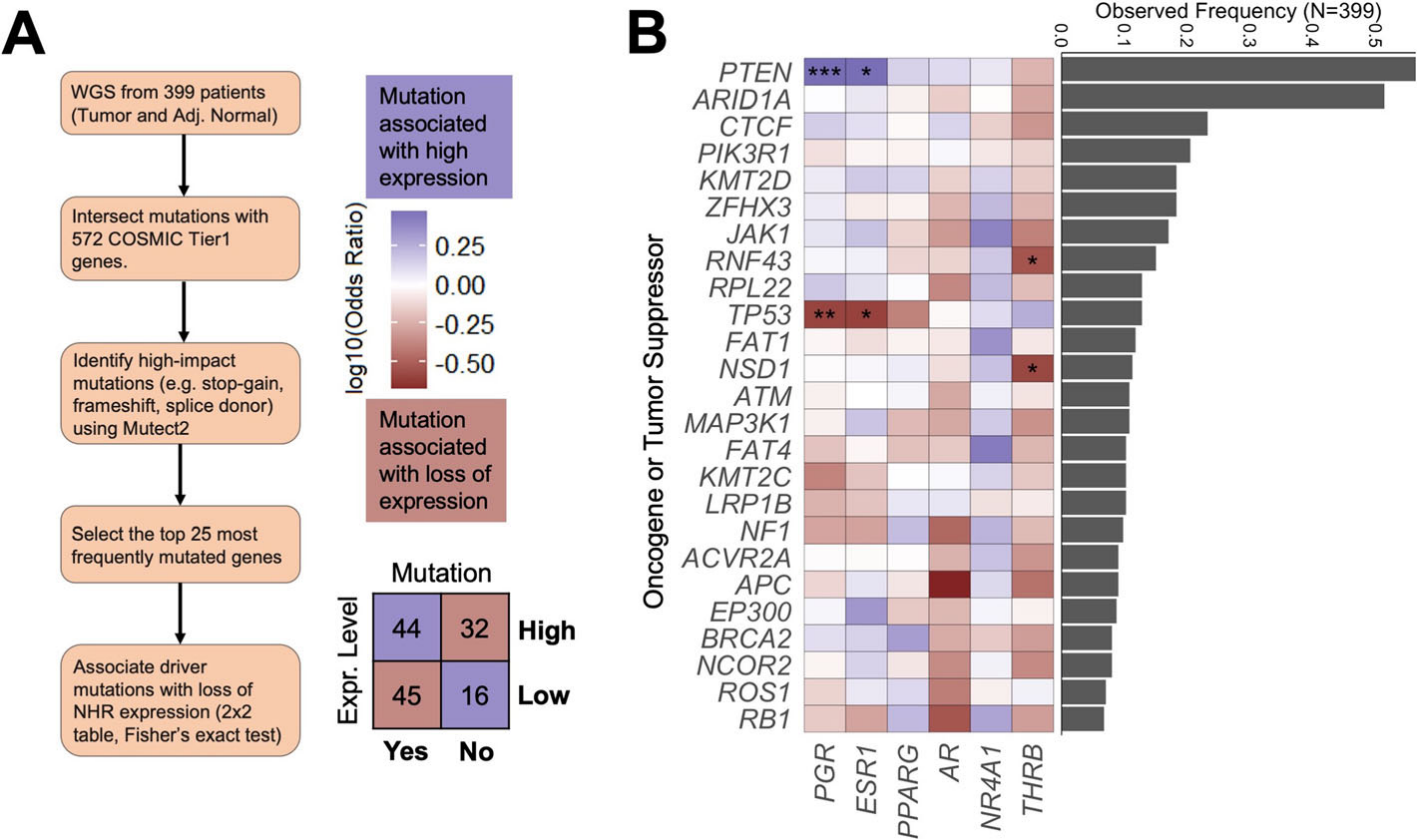
Loss of THRB expression associates with high-impact RNF43 and NSD1 mutations. **a** The steps involved for integrating TCGA data to determine the interactions between high-impact oncogenic mutations and NHR loss of expression. **b** High impact cancer-associated mutations are shown as rows, and each column corresponds to a NHR. The mutation frequency of each cancer-related mutation is shown in the bar graph on the right. Each entry in the heatmap corresponds to the odds ratio, with purple corresponding to positive associations between mutation and high expression, and red corresponding to negative associations between mutation and high expression. Asterisks within cells correspond to statistically significant odds ratios calculated using Fisher’s exact test and adjusted using the Benjamini-Hochberg method at the following thresholds: * = *q* < 0.01, ** = *q* < 0.001, *** = *q* < 0.0001

To understand the relationship between high-impact mutations in DNA sequence and loss of NHR expression, an analysis of all samples with available somatic mutation data (*N* = 399) was performed using a 2 × 2 Fisher’s exact test (Fig. 6a). The strongest relationship was found between mutations in *PTEN*, a tumor suppressor, and *PGR* expression (*OR* = 3.0, *q* = 9.1 × 10^− 5^). Furthermore, a significant relationship between the presence of *RNF43* mutations and the loss of *THRB* expression was identified (odds ratio = 0.295, *q* = 0.002) (Fig. 6b). *RNF43* encodes a ubiquitin ligase that downregulates Wnt signaling activity in pancreatic adenocarcinoma cells [25, 26]. Though the role of *RNF43* in endometrial cancer has not been previously described, endometrial cancers with increased Wnt signaling activity are associated with poor outcomes [27]. In addition, patient tumors harboring mutations in *NSD1*, a histone methyltransferase [28], are inversely associated with the loss of *THRB* expression (odds ratio = 0.269, *q* = 0.002) (Fig. 6b). Inactivating mutations in *NSD1* are associated with genomic hypomethylation and improved survival in head and neck squamous cell carcinoma [29], though no such link has been reported between *NSD1* and survival in endometrial cancer. These findings highlight novel interactions between known drivers of carcinogenesis and *THRB* expression that may form the basis for future experimentation. For instance, it would be of interest to further understand the transcriptional regulatory relationships between *THRB* and Wnt signaling, as both directly promote pro-growth transcriptional changes [30].

### Loss of *THRB* receptor expression occurs independently of *PGR* and *ESR1* expression in endometrial cancer

**Fig. 7:**
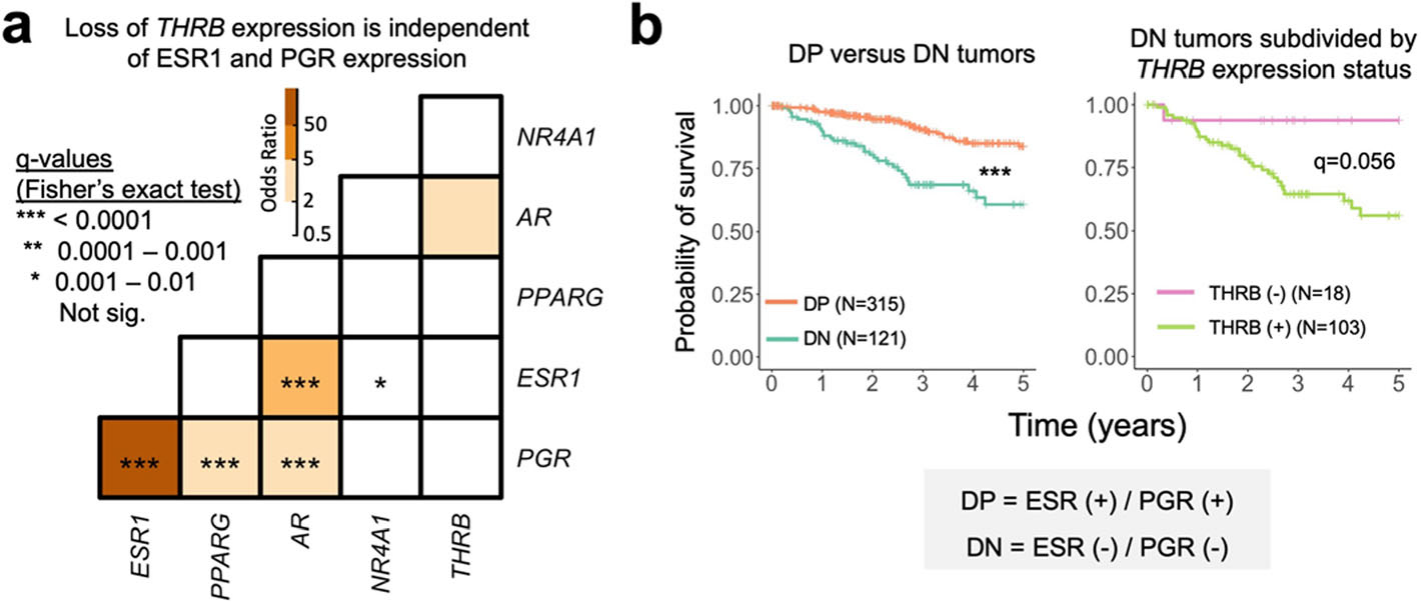
THRB is expressed independently of other NHRs and refines survival prognostication in endometrial tumors. **a** The odds ratio (calculated using Fisher’s exact test) is shown for each possible pairwise combination of the 6 NHRs. Large odds ratios (> 1) correspond to a positive interaction between any two pairs of NHRs. Asterisks within cells correspond to statistically significant odds ratios calculated using Fisher’s exact test at the following thresholds: * = *q* < 0.01, ** = *q* < 0.001, *** = *q* < 0.0001. **b** Left panel: Kaplan-Meier survival analysis between tumor subgroups defined by expression of both ESR1 and PGR (DP, for double-positive) or by the absence of expression of both ESR1 and PGR (DN, for double-negative). **b** Kaplan-Meier survival analysis of DN tumors subdivided by THRB expression status (either positive or negative for present or absent, respectively). Asterisks representing *q*-values are as described for panel **a**

Progesterone and estrogen receptor co-expression is a feature of many endometrial cancers and is associated with a favorable prognosis [10]. We wondered whether the novel NHR-based subgroups existed independently of known ESR1 and PGR-based subgroups, since this could help define novel molecular subgroups for prognostication purposes. To address this question, we first tested whether any co-expression existed between each of the 15 possible pairings between the 6 NHRs using a two-sided Fisher’s exact test (Fig. 7a). We identified the expected strong association of co-expression between *PGR* and *ESR1* (*q*-value < 2.42 × 10^− 53^ and odds ratio = 182), which is consistent with previous findings from endometrial cancer cohorts [31]. No association was present between *THRB* and any other nuclear hormone receptor, including *PGR* or *ESR1* (*q*-value > 0.01). These data demonstrate that the loss of *THRB* expression occurs independently of other well-characterized NHRs and form the basis for a novel molecular subgroup.

One potential application of this finding is that *THRB* expression could be used to refine survival prognostication models that utilize both *ESR1* and *PGR* [10]. Consistent with the established literature, endometrial cancers that lose both *ESR1* and *PGR* expression (DN, for double negative) have poor 5-year survival compared with cancers that do not lose both *ESR1* and *PGR* (DP, for double positive) (*q*-value = 1.23 × 10^− 6^, log-rank test) (Fig. 7b, left panel). However, when DN tumors are further subdivided by *THRB* expression status, DN tumors that express *THRB* have a poor prognosis (5-year probability of survival = 55.8%, Kaplan-Meier estimate), while DN tumors that do not express *THRB* have an excellent prognosis (5-year probability of survival = 93.7%, Kaplan-Meier estimate) (*q*-value = 0.056, log-rank test). Since *THRB* is expressed in a majority of DN tumors (103/121, or 85%), *THRB* could be investigated as a potential therapeutic target within a large subset of DN endometrial tumors that otherwise lack targeted treatment options and have a poor prognosis. Indeed, modulating thyroid hormone receptor beta signaling has been investigated successfully as a therapeutic strategy in murine models of hepatocellular carcinoma [32].

### Expression of miRNA-146a is associated with downregulation of *THRB* expression in endometrial cancer

**Fig. 8:**
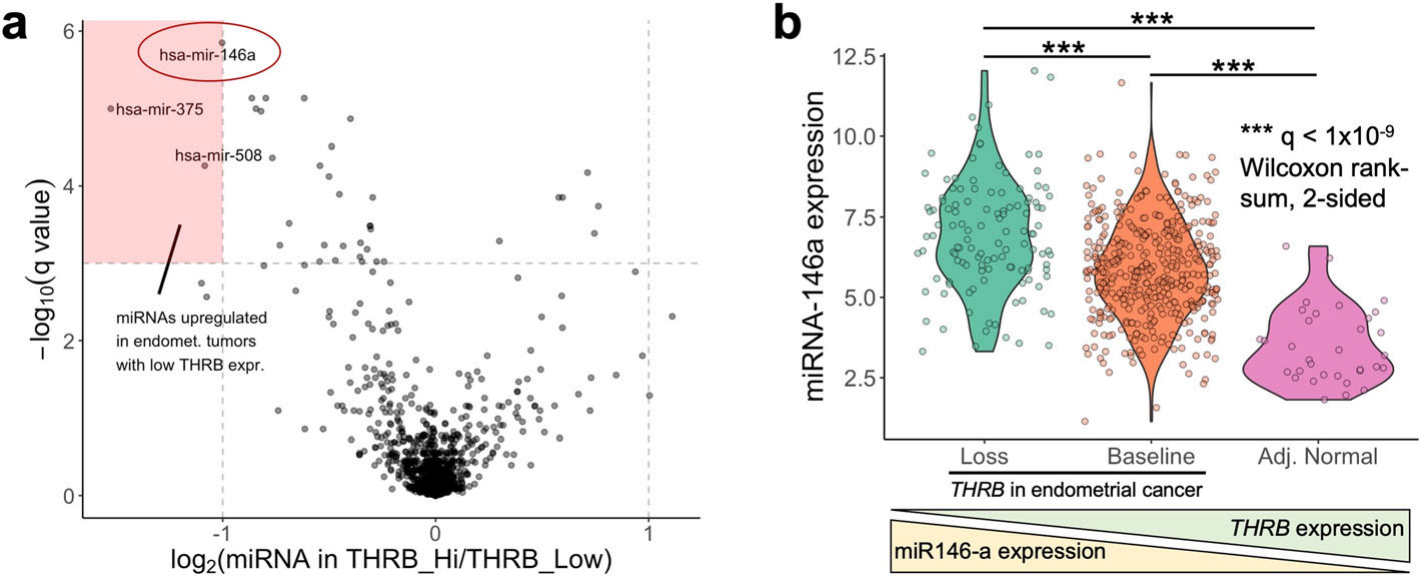
Expression of miRNA-146a is associated with downregulation of THRB in endometrial cancer. **a** The volcano plot shows the log_2_ fold change in mean miRNA expression along the x axis between tumors that maintain versus tumors that lose THRB expression. The –log_10_(*q*-value) is shown along the y axis. Each dot represents a miRNA (*N* = 1619). The expression of miRNAs toward the upper left of the plot is increased in endometrial tumors that lose THRB expression. **b** The RNA expression of miRNA-146a, in log_2_(Transcripts per million reads + 1), is shown in endometrial tumors that lose and maintain THRB expression, as well as in adjacent normal endometrial tissue

miRNAs are small RNA molecules that govern the expression of target mRNAs with complementary sequences. In cancer, the expression of miRNAs is often dysregulated, and miRNAs have been proposed as both therapeutics [33] and therapeutic targets [34] in cancer. Previous studies have established relationships between increased miRNA expression and decreased *THRB* mRNA expression in papillary thyroid carcinoma and in renal cell carcinoma [35, 36]. To determine whether there was a relationship between miRNA expression and *THRB* expression in this EC cohort, a differential miRNA expression analysis was performed between tumors with high versus low *THRB* expression. We observed 3 differentially expressed miRNAs whose expression was increased in tumors that lost *THRB* expression (q < 0.001, log_2_(|fold change|) > 1, Fig. 8a). The miRNA with the most significant *q*-value, miRNA-146a, was shown in a previous study to directly interact with *THRB* mRNA in papillary thyroid carcinoma [35]. miRNA-146a remained significantly differentially expressed when performing the same analysis only on endometrial cancers with endometrioid histology (Additional file 1). However, there were no significantly differentially expressed miRNAs among endometrial cancers with serous histology. Furthermore, miRNA-146a is expressed at relatively low levels in adjacent normal endometrial tissue (Fig. 8b). A trend is observed across both tumor and adjacent normal tissue in which decreasing levels of *THRB* are associated with increasing levels of miRNA-146a (Fig. 8b). The elevated expression of miRNA-146a in tumors that lose *THRB* expression suggests a potential biological mechanism through which *THRB* expression is lost in endometrial cancer. Future studies could also examine whether miRNA-146a itself could serve as a potential therapeutic for the high *THRB* subgroup of patients with poor 5-year survival.

## Discussion

The loss of estrogen, progesterone, and androgen receptor expression has been used to define clinically-relevant subtypes of endometrial tumors that are associated with poor outcomes [23, 31, 37]. Here, a novel open-source tool termed *receptLoss* was developed to identify subsets of patient tumors that have lost NHR expression relative to normal tissue. *ReceptLoss* relies solely on gene expression data from tumor and normal tissue and does not incorporate prior knowledge of whether an NHR has a known prognostic role. *ReceptLoss* therefore represents a novel, data-driven approach free of literature bias to identify new NHR-based biomarkers in cancer. Prior data-driven and literature-free approaches such as *oncomix* (also developed in our group) have also been developed to identify novel candidate biomarkers and driver genes in cancer based on non-standard gene expression distributions [38]. These mRNA-centric approaches provide a systematic approach for expanding the list of promising cancer biomarkers whose effects are mainly driven by changes in mRNA expression rather than by DNA mutation.

In the context of endometrial cancer, *receptLoss* correctly identified several well-known nuclear hormone receptors, including *ESR1*, *AR*, and *PGR*, that have previously been shown to have low expression in a subset of endometrial carcinomas [10, 23]. This work expands the pool of NHRs as oncologic biomarkers by showing that three previously undescribed NHRs lose expression in a subset of endometrial tumors – *THRB, PPARG*, and *NR4A1*. The previously undescribed connection between these three NHRs and endometrial cancer will be described in turn.

Thyroid receptor beta (TRβ, encoded by *THRB*) is an NHR that modulates growth signaling in both normal human development and cancerous tissue [39]. To date, the expression of *THRB* in endometrial carcinoma has not been characterized. We report, for the first time, that *THRB* expression is lost in a subset of endometrial carcinomas and is associated with poorer 5-year survival. Previous studies have examined the relationship between thyroid hormone receptor expression and outcomes in other cancers. For example, in breast cancer, TRβ protein positivity was associated with better 5-year survival in *BRCA1* mutated cancers but not in sporadic breast cancers [40].

The mechanisms by which *THRB* expression is lost in endometrial cancer remain under investigation. Here, we discuss two plausible mechanisms identified from this study. First, studies in papillary thyroid carcinoma [35] and renal cell carcinoma [36] have both shown that microRNAs downregulate expression of *THRB*, and that other molecular modifications, such as promoter methylation, are less involved in regulating *THRB* expression. We report for the first time that increased expression of miR146-a, a microRNA that has been experimentally shown to bind and degrade *THRB* mRNA in papillary thyroid carcinoma [35], is associated with the loss of *THRB* expression in endometrial carcinomas. In addition, a previous study found a single nucleotide polymorphism (rs2910164 G > C) within the pre-miRNA of miR146-a that decreases the risk for endometrial and ovarian cancer [41]. However, further studies are warranted to conclusively determine the relationship between miR146-a and *THRB* expression in endometrial cancer. Second, a relationship between microsatellite instability and loss of *THRB* expression in endometrial cancer is identified in this study for the first time. A prior study showed that mRNA expression of thyroid receptor alpha (*THRA*) is correlated with changes in intronic microsatellite length within the *THRA* locus [42]. The *THRB* locus also contains several microsatellite regions, which raises the question of whether *THRB* expression could be similarly altered by changes in intragenic microsatellite length and stability.

Intriguingly, the fact that loss of *THRB* expression is correlated with better patient survival is contrary to that of other hormone receptors, whose increased expression is often associated with better clinical outcomes. One possible explanation for this paradoxical observation is that *THRB* expression may accelerate endometrial carcinogenesis. The initial discovery of *THRB* as erbA2, an avian retroviral oncogene with a human homolog, supports this hypothesis [43, 44]. Furthermore, *THRB* is not co-expressed with other NHRs (Fig. 7a), suggesting that *THRB* expression could be used to categorize patients into novel clinically-relevant subgroups, much like the molecular expression profiling (e.g. OncotypeDx) used in breast tumors to determine whether a patient’s tumor is likely to recur [45]. These subgroups, in turn, could be useful for precision oncology efforts that modulate thyroid receptor signaling or function for the treatment of endometrial cancer. Indeed, therapeutic modulation of TRβ activity has been proposed as a therapeutic strategy to treat other types of cancer [32].

*NR4A1* is part of a small subfamily of NHRs known as orphan receptors whose activating ligands are unknown. *NR4A1* has no known associations with endometrial cancer, though previous studies have identified roles for *NR4A1* in other cancers. Specifically, two reports have supported the role of *NR4A1* as a tumor suppressor in hematologic and breast cancer [46, 47], while a third report proposed its role in potentiating TGF-β signaling and promoting metastasis in breast cancer [48]*.* In the present study, loss of *NR4A1* expression co-occurs with existing endometrial cancer subtypes, as we observed a positive relationship between *NR4A1* and *ESR1* expression (Fig. 7). Consistent with this observation, previous reports have observed the loss of *NR4A1* expression in triple-negative breast tumors, which lack expression of estrogen and progesterone receptors [47].

Our results also identified *PPARG*, a clinically-relevant NHR that encodes a protein known as peroxisome proliferator-activated receptor gamma (PPARG). PPARG is targeted by thiazolidinediones (TZDs), a class of agents used to treat type II diabetes. TZDs activate PPARG, whose natural ligands are free fatty acids, and also decrease insulin resistance [49]. This association is of interest, as obese diabetic women are at a relative risk of six for developing endometrial cancer compared with non-obese, non-diabetic women [50]. TZDs have been touted as potential anti-cancer agents, and differences in the expression of *PPARG* within tumors could aid in the personalization of therapeutic regimens [51]. The intersection between TZD administration, *PPARG* expression, and endometrial cancer development deserves additional study.

## Conclusions

In summary, we develop an open-source software package, termed *receptLoss*, to identify novel subgroups of endometrial cancer patients based on patterns of nuclear hormone receptor expression between tumor and adjacent normal tissue. *ReceptLoss* correctly identified established patterns of NHR expression and detected 3 NHRs whose expression loss had not been described in endometrial cancer. The previously unreported observation that *THRB* is lost in a subset of endometrial cancers and is associated with better 5-year survival could aid in the development of prognostic biomarkers and of targeted therapeutic regimens for endometrial carcinoma that modulate thyroid receptor signaling. More broadly, *receptLoss* can be utilized to identify changes in NHRs in additional cancer types where gene expression datasets from both tumor and normal tissue are available.

## Supplementary information

**Additional file 1.**

## Data Availability

All data is freely available via the Genomic Data Commons. A computationally-reproducible workflow with the code used to download the data and perform the analysis are available in Additional file 1.

## Abbreviations

DN: Endometrial carcinomas doubly negative for ESR1 and PGR expression
DP: Endometrial carcinomas doubly positive for ESR1 and PGR expression
EC: Endometrial cancer
ER: Estrogen receptor
FPKM: Fragments per kilobase per million reads
GDC: Genomic data commons
NHR: Nuclear hormone receptor
PGR: Progesterone receptor
TCGA: The Cancer Genome Atlas
THRB: Thyroid Hormone Receptor Beta
TZD: Thiazolidinediones

## References

[1] Alexander SP, Christopoulos A, Davenport AP, Kelly E, Marrion NV, Peters JA, et al. The concise guide to pharmacology 2017/18: Nuclear hormone receptors. Br J Pharmacol. 2017(174):S208–24. 10.1111/bph.13880 PMC5650662 29055032

[2] Rivera R, Yacobson I, Grimes D. The mechanism of action of hormonal contraceptives and intrauterine contraceptive devices. Am J Obstet Gynecol. 1999;181(5):1263–9. 10.1016/s0002-9378(99)70120-1 10561657

[3] Krassas GE, Pontikides N, Kaltsas T, Papadopoulou P, Paunkovic J, Paunkovic N (1999). Disturbances of menstruation in hypothyroidism. Clin Endocrinol.

[4] Kakuno Y, Amino N, Kanoh M, Kawai M, Fujiwara M, Kimura M (2010). Menstrual disturbances in various thyroid diseases. Endocr J.

[5] Islami F, Goding Sauer A, Miller KD, Siegel RL, Fedewa SA, Jacobs EJ (2018). Proportion and number of cancer cases and deaths attributable to potentially modifiable risk factors in the United States. CA Cancer J Clin.

[6] Lortet-Tieulent J, Ferlay J, Bray F, Jemal A (2018). International patterns and trends in endometrial Cancer incidence, 1978–2013. JNCI J Natl Cancer Inst.

[7] Smith D, Stewart CJR, Clarke EM, Lose F, Davies C, Armes J (2017). ER and PR expression and survival after endometrial cancer. Gynecol Oncol.

[8] Bokhman JV (1983). Two pathogenetic types of endometrial carcinoma. Gynecol Oncol.

[9] Getz G, Gabriel SB, Cibulskis K, Lander E, Sivachenko A, Sougnez C (2013). Integrated genomic characterization of endometrial carcinoma. Nature..

[10] Kleine W, Maier T, Geyer H, Pfleiderer A (1990). Estrogen and progesterone receptors in endometrial cancer and their prognostic relevance. Gynecol Oncol.

[11] Backes FJ, Walker CJ, Goodfellow PJ, Hade EM, Agarwal G, Mutch D (2016). Estrogen receptor-alpha as a predictive biomarker in endometrioid endometrial cancer. Gynecol Oncol.

[12] Hanekamp EE, Gielen SCJP, Smid-koopman E, De Ruiter PE, Chadha-ajwani S, Brinkmann AO (2003). Consequences of loss of progesterone receptor expression in development of invasive endometrial Cancer. Clin Cancer Res.

[13] Pique D, Greally J, Mar J. receptLoss: unsupervised identification of genes with expression loss in subsets of tumors: Bioconductor; 2020. R package: Version 1.0.0.

[14] Creasman WT (1993). Prognostic significance of hormone receptors in endometrial cancer. Cancer..

[15] Zhang Y, Zhao D, Gong C, Zhang F, He J, Zhang W (2015). Prognostic role of hormone receptors in endometrial cancer: a systematic review and meta-analysis. World J Surg Oncol.

[16] Grossman RL, Heath AP, Ferretti V, Varmus HE, Lowy DR, Kibbe WA (2016). Toward a shared vision for Cancer genomic data. N Engl J Med.

[17] Institute NC. RNA-seq quantification [Internet].

[18] Therneau TM. A package for survival analysis in S: CRAN; 2015. Version 2.

[19] Forbes SA, Beare D, Gunasekaran P, Leung K, Bindal N, Boutselakis H (2014). COSMIC: exploring the world’s knowledge of somatic mutations in human cancer. Nucleic Acids Res.

[20] Cibulskis K, Lawrence MS, Carter SL, Sivachenko A, Jaffe D, Sougnez C (2013). Sensitive detection of somatic point mutations in impure and heterogeneous cancer samples. Nat Biotechnol.

[21] Benjamini Y, Hochberg Y (1995). Controlling the false discovery rate: a practical and powerful approach to multiple testing. J R Stat Soc B.

[22] Mar JC. The rise of the distributions: why non-normality is important for understanding the transcriptome and beyond. Biophys Rev. 2019:89–94. 10.1007/s12551-018-0494-4 PMC6381358 30617454

[23] Zadeh SL, Duska LR, Mills AM (2017). Androgen receptor expression in endometrial carcinoma. Int J Gynecol Pathol.

[24] Hashmi AA, Hussain ZF, Qadri A, Irfan M, Ramzan S, Faridi N (2018). Androgen receptor expression in endometrial carcinoma and its correlation with clinicopathologic features. BMC Res Notes.

[25] Jiang X, Hao H-X, Growney JD, Woolfenden S, Bottiglio C, Ng N (2013). Inactivating mutations of RNF43 confer Wnt dependency in pancreatic ductal adenocarcinoma. Proc Natl Acad Sci.

[26] Tsukiyama T, Fukui A, Terai S, Fujioka Y, Shinada K, Takahashi H (2015). Molecular role of RNF43 in canonical and noncanonical Wnt signaling. Mol Cell Biol.

[27] Liu Y, Patel L, Mills GB, Lu KH, Sood AK, Ding L (2014). Clinical significance of CTNNB1 mutation and Wnt pathway activation in endometrioid endometrial carcinoma. J Natl Cancer Inst.

[28] Qiao Q, Li Y, Chen Z, Wang M, Reinberg D, Xu RM (2011). The structure of NSD1 reveals an autoregulatory mechanism underlying histone H3K36 methylation. J Biol Chem.

[29] Bui N, Huang JK, Bojorquez-Gomez A, Licon K, Sanchez KS, Tang SN (2018). Disruption of NSD1 in head and neck cancer promotes favorable chemotherapeutic responses linked to hypomethylation. Mol Cancer Ther.

[30] Skah S, Uchuya-Castillo J, Sirakov M, Plateroti M (2017). The thyroid hormone nuclear receptors and the Wnt/β-catenin pathway: an intriguing liaison. Dev Biol.

[31] Tomica D, Rami S, Danoli D, Kne F, Kolak T (2014). A Correlation between the Expression of Estrogen Receptors and Progesterone Receptors in Cancer Cells and in the Myometrium and Prognostic Factors in Endometrial. Cancer.

[32] Puliga E, Min Q, Tao J, Zhang R, Pradhan-Sundd T, Poddar M (2017). Thyroid hormone receptor-β agonist GC-1 inhibits met-β-catenin–driven hepatocellular Cancer. Am J Pathol.

[33] Rupaimoole R, Slack FJ (2017). MicroRNA therapeutics: towards a new era for the management of cancer and other diseases. Nat Rev Drug Discov.

[34] Wen D, Danquah M, Chaudhary AK, Mahato RI (2015). Small molecules targeting microRNA for cancer therapy: promises and obstacles. J Control Telease.

[35] Jazdzewski K, Boguslawska J, Jendrzejewski J, Liyanarachchi S, Pachucki J, Wardyn KA (2011). Thyroid hormone receptor β (THRB) is a major target gene for microRNAs deregulated in papillary thyroid carcinoma (PTC). J Clin Endocrinol Metab.

[36] Wojcicka A, Piekielko-Witkowska A, Kedzierska H, Rybicka B, Poplawski P, Boguslawska J (2014). Epigenetic regulation of thyroid hormone receptor beta in renal cancer. PLoS One.

[37] Tangen IL, Werner HMJ, Berg A, Halle MK, Kusonmano K, Trovik J (2014). Loss of progesterone receptor links to high proliferation and increases from primary to metastatic endometrial cancer lesions. Eur J Cancer.

[38] Piqué DG, Montagna C, Greally JM, Mar JC (2019). A novel approach to modelling transcriptional heterogeneity identifies the oncogene candidate CBX2 in invasive breast carcinoma. Br J Cancer.

[39] Ortiga-Carvalho TM, Sidhaye AR, Wondisford FE (2014). Thyroid hormone receptors and resistance to thyroid hormone disorders. Nat Rev Endocrinol.

[40] Heublein S, Mayr D, Meindl A, Angele M, Gallwas J, Jeschke U (2015). Thyroid hormone receptors predict prognosis in BRCA1 associated breast cancer in opposing ways. PLoS One.

[41] Liu X, Xu B, Li S, Zhang B, Mao P, Qian B (2015). Association of SNPs in miR-146a, miR-196a2, and miR-499 with the risk of endometrial/ovarian cancer. Acta Biochim Biophys Sin Shanghai.

[42] Onda M, Li D, Suzuki S, Nakamura I, Takenoshita S, Brogren CH (2002). Expansion of microsatellite in the thyroid hormone receptor-α1 gene linked to increased receptor expression and less aggressive thyroid cancer. Clin Cancer Res.

[43] Rider SH, Gorman PA, Shipley JM, Moore G, Vennstromt B, Solomon E (1987). Localization of the oncogene c-erbA2 to human chromosome 3. Ann Hum Genet.

[44] Jansson M, Philipson L, Vennström B (1983). Isolation and characterization of multiple human genes homologous to the oncogenes of avian erythroblastosis virus. EMBO J.

[45] Cronin M, Sangli C, Liu M-L, Pho M, Dutta D, Nguyen A (2007). Analytical validation of the Oncotype DX genomic diagnostic test for recurrence prognosis and therapeutic response prediction in node-negative, estrogen receptor-positive breast cancer. Clin Chem.

[46] Wenzl K, Troppan K, Neumeister P, Deutsch AJA (2015). The nuclear orphan receptor NR4A1 and NR4A3 as tumor suppressors in hematologic neoplasms. Curr Drug Targets.

[47] Wu H, Bi J, Peng Y, Huo L, Yu X, Yang Z (2017). Nuclear receptor NR4A1 is a tumor suppressor down-regulated in triple-negative breast cancer. Oncotarget..

[48] Zhou FF, Drabsch Y, Dekker TJA, De Vinuesa AG, Li Y, Hawinkels LJAC, et al. Nuclear receptor NR4A1 promotes breast cancer invasion and metastasis by activating TGF-β signalling. Nat Commun. 2014;5. 10.1038/ncomms4388 24584437

[49] Quinn C, Hamilton P, Lockhart C, Mcveigh G (2008). Thiazolidinediones: effects on insulin resistance and the cardiovascular system. Br J Pharmacol.

[50] Friberg E, Mantzoros CS, Wolk A (2007). Diabetes and risk of endometrial cancer: a population-based prospective cohort study. Cancer Epidemiol Biomark Prev.

[51] Blanquicett C, Roman J, Hart CM (2008). Thiazolidinediones as anti-cancer agents. Cancer Ther.

