# Supplementary material for: Identification of a novel subgroup of endometrial cancer patients with loss of thyroid hormone receptor beta expression and improved survival
Source: BMC Cancer. 2020 Sep 7;20:857. doi: 10.1186/s12885-020-07325-y (PMC7487950; doi:10.1186/s12885-020-07325-y)
Supplement: Supplementary file 1 — Additional file 1. [file 12885_2020_7325_MOESM1_ESM.html]

Identification of a novel subgroup of endometrial cancer patients with loss of thyroid hormone receptor expression and improved survival


# Identification of a novel subgroup of endometrial cancer patients with loss of thyroid hormone receptor expression and improved survival

## Identification of a novel subgroup of endometrial cancer patients with loss of thyroid hormone receptor expression and improved survival

- 1 Introduction
- 2 Setup and download data
  - 2.1 Load in the libraries
  - 2.2 Download and filter datasets
  - 2.3 Load and clean the endometrial cancer expression data
  - 2.4 Perform PCA and look for hospital and sequencing plate bias
  - 2.5 Check and fix BMI clinical data in endometrial cancer
  - 2.6 Print out the clinical data
  - 2.7 Load the endometrial cancer microRNA data
  - 2.8 Load the nuclear hormone receptor database
- 3 Identify NHRs with expression loss in a subset of tumors
  - 3.1 Classify tumors into low or high expression groups using boundary
  - 3.2 Check for technical bias by sequencing plate for the 6 genes.
  - 3.3 Check for technical bias by sequencing plate for all genes.
- 4 Identify survival differences based on NHR expression
  - 4.1 Identify clinical features of THRB-low subtype
  - 4.2 Look at survival differences between endometroid tumors
  - 4.3 Look at survival differences between serous tumors
- 5 Examine combinatorial relationships between survival groups
- 6 Test the association between NHR expression and mutations
  - 6.1 Cluster hormone receptor and mutations status
  - 6.2 Statistical testing (Fisher’s exact) between NHR expression and mutation
  - 6.3 Calculate Odds Ratio between each *pair* of genes
- 7 Differential microRNA expression analysis
  - 7.1 Volcano plot showing miRNA expression
  - 7.2 Volcano plot showing miRNA differential expression in tumors with endometrioid histology
  - 7.3 Volcano plot showing miRNA differential expression in tumors with serous histology
  - 7.4 Expression of miRNA-146a across various tissue types
  - 7.5 Are the THRB-low tumors the same as the Type I endometrial cancers?
- 8 THRB expression in other cancers
  - 8.1 What does THRB receptor expression look like in other cancers?
  - 8.2 Run receptLoss on other cancer types and look at THRB expression here
  - 8.3 Is there a difference in survival between high and low groups based on THRB expression in other tumors? Classify the tumors first, then perform K-M survival analysis
- 9 Session Information
  - 9.1 Supplementary scripts

Daniel G. Piqué, John M. Greally, and Jessica C. Mar

June 24, 2020

# 1 Introduction

# 2 Setup and download data

## 2.1 Load in the libraries

```
#library(TCGAbiolinks)  #uncomment if running for first time
library(SummarizedExperiment)
library(RColorBrewer)
library(here)
library(mclust)
library(biomaRt)
library(tidyverse)
library(janitor)
library(survival)
library(ggdendro)
library(limma)
library(fgsea)
library(ggrepel)
library(patchwork)
library(receptLoss)
source(here("scripts", "2019_endomet_smry_suppl.R"))
```

## 2.2 Download and filter datasets

**mRNA expression**

- TCGA-UCEC - Endometrial adenocarcinoma
- TCGA-BRCA - Breast adenocarcinoma
- TCGA-LUAD - Lung squamous adenocarcinoma
- TCGA-LUSC - Lung squamous cell carcinoma
- TCGA-OV - Ovarian adenocarcinoma

**microRNA expression**

- TCGA-UCEC - Endometrial adenocarcinoma

We use the R package `TCGAbiolinks` to download the expression and clinical data from these cancer types in GDC data release version 13.0.

```
project_types <- c("TCGA-UCEC", "TCGA-BRCA", "TCGA-LUAD", "TCGA-LUSC")

if(!file.exists(here("data", paste0(project_types, "-exp.rds")))){
  ## download the data for each of the 5 cancer types
  ## uncomment the GDCdownload and saveRDS lines if running for first time
  eq <- GDCquery(project = project_types,
                  data.category = c("Transcriptome Profiling"),
                  data.type = c("Gene Expression Quantification"),
                  workflow.type = "HTSeq - FPKM")
  #GDCdownload(eq)
  
  ## prepare and save the datasets
  for(i in 1:length(project_types)){
    eq <- GDCquery(project = project_types[i],
                  data.category = c("Transcriptome Profiling"),
                  data.type = c("Gene Expression Quantification"),
                  workflow.type = "HTSeq - FPKM")
    #GDCdownload(eq)
    e <- GDCprepare(eq, save=FALSE)
    #saveRDS(e, file=here("data",paste0(project_types[i], "-exp.rds")))
    print(project_types[i])
  }
  
  #prepare and save the miRNA datasets
  mi <- GDCquery(project = "TCGA-UCEC",
                 workflow.type = "BCGSC miRNA Profiling",
                 data.category = c("Transcriptome Profiling"),
                 data.type = c("miRNA Expression Quantification"))
  #GDCdownload(mi)
  m <- GDCprepare(mi, save=FALSE)
  #saveRDS(m, file=here("data", "TCGA-UCEC-mirna.rds"))

}
```

## 2.3 Load and clean the endometrial cancer expression data

```
e <- readRDS(here("data", "TCGA-UCEC-exp.rds")) %>% clean_dataset()
```

```
## [1] 56716   587
## [1] "Dimensions after FFPE removed:"
## [1] 56716   583
## [1] "Dimensions of tumor and adj nml dataframes:"
## [1] 56716   547
## [1] 56716    35
## [1] "Dimensions of tumor and adj nml dataframes (duplicates removed):"
## [1] 56716   543
## [1] 56716    35
## [1] "Dimensions of tumor and adj nml dataframes (duplicates removed + pts with NA values removed):"
## [1] 56716   543
## [1] 56716    35
## [1] "Dimensions of tumor and adj nml dataframes (duplicates removed + pts with NA values removed + 20% filter):"
## [1] 37083   543
## [1] 37458    35
## [1] "Dimensions of tumor and adj nml dataframes (duplicates removed + pts with NA values removed + 20% filter + intersection):"
## [1] 35308   543
## [1] 35308    35
```

```
en <- e$en
e <- e$et
  
esr1 = "ENSG00000091831"
ar = "ENSG00000169083"
pr = "ENSG00000082175"
```

## 2.4 Perform PCA and look for hospital and sequencing plate bias

```
pca <- prcomp(t(assay(e)), center = T, scale. = T)
```

### 2.4.1 Add centroids (one for each hospital) overlayed on samples

```
pca.centroids <- aggregate(data.frame(pca$x)[,1:10], list(Type = colData(e)$hosp), mean)

pca.m.hosp <- reshape2::melt(cbind(data.frame(pca$x), hosp=colData(e)$hosp),
                             id.vars=c("PC1", "PC2","hosp"))
pca.centroids.hosp <- aggregate(data.frame(pca$x)[,1:10], list(Type = colData(e)$hosp), mean)
pca.centroids.hosp.m <- reshape2::melt(pca.centroids.hosp, id.vars=c("Type","PC1", "PC2"))

ggplot(pca.centroids.hosp.m, aes(x=PC1, y=PC2, color=Type)) + 
  geom_point(data=pca.m.hosp, aes(PC1, PC2, color=hosp, alpha=0.2), 
             alpha=0.1, size=0.5) +
  centroid_plot_elem()
```

### 2.4.2 Add centroids (one for each sequencing plate) overlayed on samples

```
pca.m.plate <- reshape2::melt(cbind(data.frame(pca$x), seqPlate=colData(e)$seqPlate), 
                              id.vars=c("PC1", "PC2","seqPlate"))
pca.centroids.plate <- aggregate(data.frame(pca$x)[,1:10], list(Type = colData(e)$seqPlate), mean)
pca.centroids.plate.m <- reshape2::melt(pca.centroids.plate, id.vars=c("Type","PC1", "PC2"))

ggplot(pca.centroids.plate.m, aes(x=PC1, y=PC2, color=Type)) + 
  geom_point(data=pca.m.plate, aes(PC1, PC2, color=seqPlate, alpha=0.2),
             alpha=0.1, size=0.5) + 
  centroid_plot_elem()
```

We can see that there are no centroids that are visible outliers among either hospitals or sequencing plate. This suggests that the variability observed in the sequencing data is not due to differences in hospitals where the samples were taken from or to differences in the plate that each sample was sequenced on.

## 2.5 Check and fix BMI clinical data in endometrial cancer

```
colData(e)$bmi <- colData(e)$weight / (colData(e)$height/100)^2
plot(colData(e)$weight, colData(e)$height)
```

```
#are there any bmi outliers?
hist(colData(e)$bmi)
```

```
bmiOutlIdx <- which(colData(e)$bmi > threeSdAboveMean(mean(colData(e)$bmi, na.rm=T), sd = sd(colData(e)$bmi, na.rm=T)))

#are there any height outliers?
hist(colData(e)$height)
```

```
heightOutlIdx <- which(colData(e)$height < threeSdBelowMean(mean(colData(e)$height, na.rm=T), sd = sd(colData(e)$height, na.rm=T)))
#this looks like it may have been entered in inches. 66 inches is 5 1/2 feet - more likely than 66 cm, which is more likely than 2.16 feet!

#hist(colData(e)$weight)
weightOutlIdx <- which(colData(e)$weight > threeSdAboveMean(mean(colData(e)$weight, na.rm=T), sd = sd(colData(e)$weight, na.rm=T)))

#if a patient is both a bmi outlier and weight (extremely above) 
#or height (extremely below) outlier, assume that they are incorrectly 
#entered in English units (not metric).
weightOutl <- intersect(weightOutlIdx, bmiOutlIdx)
heightOutl <- intersect(heightOutlIdx, bmiOutlIdx)

colData(e)[weightOutl,c("weight", "height")] #weight is likely in pounds
```

```
## DataFrame with 1 row and 2 columns
##                                 weight    height
##                              <numeric> <numeric>
## TCGA-BG-A0M8-01A-12R-A104-07       209       160
```

```
colData(e)[heightOutl,c("weight", "height")] #height is likely in inches
```

```
## DataFrame with 1 row and 2 columns
##                                 weight    height
##                              <numeric> <numeric>
## TCGA-SJ-A6ZI-01A-12R-A34R-07        93        66
```

```
colData(e)[weightOutl,"weight"]  <- poundToKg(colData(e)[weightOutl,"weight"])
colData(e)[heightOutl,"height"]  <- inToCm(colData(e)[heightOutl,"height"])

plot(colData(e)$weight, colData(e)$height)
```

```
#recompute bmi with new values
colData(e)$bmi <- colData(e)$weight / (colData(e)$height/100)^2
```

### 2.5.1 Normalize the clinical data

```
days_last_fu <- ifelse(is.na(colData(e)$days_to_death), colData(e)$days_to_last_follow_up,colData(e)$days_to_death)
days_last_fu <- ifelse(days_last_fu < 1, 1, days_last_fu)
days_last_fu <- ifelse(days_last_fu > 365.25 * 5, 365.25 * 5, days_last_fu)

vital_stat <- ifelse(colData(e)$vital_status == "alive", 0, 1)
vital_stat <- ifelse(days_last_fu >= 365.25 * 5, 0, vital_stat)

colData(e)$days_last_fu_5yr <- days_last_fu
colData(e)$vital_stat_5yr <- vital_stat
colData(e)$bmi <- colData(e)$weight / (colData(e)$height/100)^2

#remove the patient with missing survival data
e <- e[,!is.na(colData(e)$vital_stat_5yr)]

datAddMissing <- function(colDat){
  #convert to character, add missing label
  cd <- as.character(colDat)
  cd[is.na(cd)] <- "Missing"
  return(cd)
}

colData(e)$subtype_mrna_expression_cluster <- datAddMissing(colData(e)$subtype_mrna_expression_cluster)
colData(e)$subtype_IntegrativeCluster <- datAddMissing(colData(e)$subtype_IntegrativeCluster)
colData(e)$subtype_msi_status_7_marker_call <- datAddMissing(colData(e)$subtype_msi_status_7_marker_call)
colData(e)$subtype_cna_cluster_k4 <- datAddMissing(colData(e)$subtype_cna_cluster_k4)
colData(e)$subtype_X2009stagegroup <- datAddMissing(colData(e)$subtype_X2009stagegroup)
colData(e)$subtype_histology <- datAddMissing(colData(e)$subtype_histology)
colData(e)$subtype_tumor_grade <- datAddMissing(colData(e)$subtype_tumor_grade)
colData(e)$subtype_ethnicity <- datAddMissing(colData(e)$subtype_ethnicity)
colData(e)$race <- datAddMissing(colData(e)$race)
colData(e)$seqPlate <- datAddMissing(colData(e)$seqPlate)
colData(e)$hosp <- datAddMissing(colData(e)$hosp)

subtype_mrna_mm <- model.matrix( ~ subtype_mrna_expression_cluster - 1, data=colData(e) )
subtype_integ_mm <- model.matrix( ~ subtype_IntegrativeCluster - 1, data=colData(e) )
subtype_msi_mm <- model.matrix( ~ subtype_msi_status_7_marker_call - 1, data=colData(e) )
subtype_cna_k4_mm <- model.matrix( ~ subtype_cna_cluster_k4 - 1, data=colData(e) )
stage <- model.matrix( ~ subtype_X2009stagegroup - 1, data=colData(e) )
histo <- model.matrix( ~ subtype_histology - 1, data=colData(e) )
grade <- model.matrix( ~ subtype_tumor_grade - 1, data=colData(e) )
race_mm <- model.matrix( ~ race - 1, data=colData(e) )
ethnicity_mm <- model.matrix( ~ subtype_ethnicity - 1, data=colData(e))
seqplate_mm <- model.matrix( ~ seqPlate - 1, data=colData(e))
hosp_mm <- model.matrix( ~ hosp - 1, data=colData(e))


normClinDat <- data.frame(cbind(subtype_mrna_mm, subtype_integ_mm, subtype_msi_mm, subtype_cna_k4_mm, stage, histo, grade, race_mm, ethnicity_mm, seqplate_mm, hosp_mm))
#remove redundant variables
duplicated.columns <- duplicated(t(normClinDat))
normClinDat <- normClinDat[, !duplicated.columns]

#replace continuous variables with the mean
cont_vars <- c("weight", "height", "days_to_birth", "days_last_fu_5yr", "bmi")
for(i in 1:ncol(colData(e)[,cont_vars])){
  colData(e)[,cont_vars][is.na(colData(e)[,cont_vars][,i]), i] <- median(colData(e)[,cont_vars][,i], na.rm = TRUE)
}
#replace colData with the median
#fix height issue - someone probably entered in height in feet, causing a really large BMI. Replace this by either converting to inches or replacing with the colMean (treat as missing)

rescaledDat <- data.frame(scale(colData(e)[,c("weight", "height", "days_to_birth", "bmi")]))
unscaledDat <- data.frame(colData(e)[,c("weight", "height", "days_to_birth", "bmi")])
normClinDat <- cbind(normClinDat, unscaledDat, colData(e)[,c("days_last_fu_5yr", "vital_stat_5yr")])

#remove the patient for which we have no survival information on them?
#add columns for age categories
```

## 2.6 Print out the clinical data

```
age <- {colData(e)$days_to_birth / -365.25} 
age %>% summary()
```

```
##    Min. 1st Qu.  Median    Mean 3rd Qu.    Max. 
##   31.85   57.45   64.20   64.47   71.65   90.00
```

```
age %>% sd()
```

```
## [1] 11.09085
```

```
race <- colData(e)$race
table(race)
```

```
## race
##          american indian or alaska native 
##                                         4 
##                                     asian 
##                                        20 
##                 black or african american 
##                                       106 
## native hawaiian or other pacific islander 
##                                         9 
##                              not reported 
##                                        32 
##                                     white 
##                                       371
```

```
ethnicity <- colData(e)$ethnicity
table(ethnicity)
```

```
## ethnicity
##     hispanic or latino not hispanic or latino           not reported 
##                     15                    372                    155
```

```
histo <- colData(e)$subtype_histology
table(histo)
```

```
## histo
## Endometrioid      Missing        Mixed       Serous 
##          304          172           13           53
```

```
stage <- colData(e)$subtype_X2009stagegroup
table(stage)
```

```
## stage
##   Missing   Stage I  Stage II Stage III  Stage IV   Unknown 
##       172       251        25        74        17         3
```

```
surv_5yr <- colData(e)$vital_stat_5yr
table(surv_5yr)
```

```
## surv_5yr
##   0   1 
## 460  82
```

## 2.7 Load the endometrial cancer microRNA data

```
rpm_str <- "reads_per_million_miRNA_mapped_"
mir <- readRDS(file=here("data", "TCGA-UCEC-mirna.rds")) %>% 
  as_tibble %>%
  dplyr::select(miRNA_ID, starts_with("reads_per_million_miRNA_mapped")) %>%
  rename_at(vars(-miRNA_ID), funs(substr(., nchar(rpm_str)+1, nchar(.)))) %>%
  filter(rowSums(.[,2:ncol(.)]) != 0)

sum(rowSums(mir[,2:ncol(mir)]) == 0)
```

```
## [1] 0
```

```
mir_t <- mir %>% 
  dplyr::select(which(substr(names(.), 14, 15) == "01")) %>%
  dplyr::select(which(!duplicated(substr(names(.), 1, 12)))) %>%
  dplyr::select(which(substr(names(.),1,12) %in% substr(colnames(e), 1,12)))

mir_n <- mir %>% dplyr::select(which(substr(names(.), 14, 15) == "11"))
```

## 2.8 Load the nuclear hormone receptor database

```
geneMap <- read_csv(here("data", "ucsc_map", "geneMap.txt")) %>% mutate(ucsc = substr(ucsc, 1,8))

nuclReceptDb <- read_csv(file=here("data", "nucl_recept_db", "targets_and_families.csv")) %>% clean_names()
## nuclReceptDb <- read.csv(url("http://www.guidetopharmacology.org/DATA/targets_and_families.csv"), header=T)
nuclReceptDb_sbst <- nuclReceptDb[nuclReceptDb$type == "nhr",]

nuclReceptDb_sbst_ensg <- nuclReceptDb_sbst %>% 
  left_join(geneMap %>% 
              dplyr::select(-ucsc, -hgnc_symbol) %>% 
              distinct, by=c("human_entrez_gene" = "entrezgene"))
```

# 3 Identify NHRs with expression loss in a subset of tumors

The following shows the numbers for the workflow.

```
filtCutoffLower <- 0.2

res <- receptLoss::receptLoss(exprMatrNml=assay(en), exprMatrTum=assay(e), nSdBelow=2, minPropPerGroup=filtCutoffLower)

#Select NHRs with rankings 1 sd above mean of all other genes. 
diffMeanSd <- mean(res %>% filter(meetsMinPropPerGrp) %>% pull(deltaMu)) + sd(res %>% filter(meetsMinPropPerGrp) %>% pull(deltaMu))

res2 <- res %>% mutate(is_nuclr_recpt = geneNm %in% nuclReceptDb_sbst_ensg$ensembl_gene_id) %>%
  left_join(nuclReceptDb_sbst_ensg, by=c("geneNm" = "ensembl_gene_id")) %>% 
  mutate(top_genes = is_nuclr_recpt & meetsMinPropPerGrp & deltaMu > diffMeanSd,
         oneSdAbove = lowerBound > 0 & meetsMinPropPerGrp & deltaMu > diffMeanSd) 

#unique(nuclReceptDb_sbst_ensg$ensembl_gene_id) %>% .[!is.na(.)] %>% length()
## Total number of:
## genes
nrow(res2)
```

```
## [1] 35308
```

```
## NHR ENSG ids
unique(nuclReceptDb_sbst_ensg$ensembl_gene_id) %>% .[!is.na(.)] %>% length()
```

```
## [1] 53
```

```
## NHRs that overlap with genes that are expressed (selected after initial filt)
filter(res2, is_nuclr_recpt) %>% nrow()
```

```
## [1] 47
```

```
## sum of all genes with boundary greater than 0
sum(res2$lowerBound > 0)
```

```
## [1] 17034
```

```
## sum of all nhrs with boundary greater than 0
sum({filter(res2, is_nuclr_recpt) %>% pull(lowerBound)} > 0)
```

```
## [1] 33
```

```
## sum of all genes with boundary greater than 0 & > 20% samples per group
filter(res2, lowerBound > 0, meetsMinPropPerGrp) %>% nrow()
```

```
## [1] 6536
```

```
## sum of all nhrs with boundary greater than 0 & > 20% samples per group
filter(res2, is_nuclr_recpt, lowerBound > 0, meetsMinPropPerGrp) %>% nrow()
```

```
## [1] 21
```

```
## number of genes (including nhrs) that are 1 sd above mean deltaMu value
filter(res2, oneSdAbove) %>% nrow()
```

```
## [1] 896
```

```
## number of nhrs that are 1 sd above mean deltaMu value
top_nhrs <- filter(res2, is_nuclr_recpt, oneSdAbove)# %>% nrow()
nrow(top_nhrs)
```

```
## [1] 6
```

```
## plot
set2 <- RColorBrewer::brewer.pal(8,"Set2")
set3 <- RColorBrewer::brewer.pal(12,"Set3")

delta_mu_plot <- ggplot(res2 %>% filter(meetsMinPropPerGrp, is_nuclr_recpt), aes(x=deltaMu, y = ..density..)) + 
  geom_histogram(fill=set3[5], color=set3[5], alpha=0.5, bins=40) + 
  geom_vline(xintercept = diffMeanSd, linetype='dashed') + 
  theme_classic() + 
  geom_histogram(data=res2 %>% filter(meetsMinPropPerGrp), aes(x=deltaMu, y = ..density..), 
                 fill=set3[4], color=set3[4], alpha=0.2, bins=40) + 
  ggHist + xlab("") + ylab("")
delta_mu_plot
```

```
ggsave(filename = here("output", "delta_mu_plot.png"), plot = delta_mu_plot, width = 8, height = 5, units = "in")
```

## 3.1 Classify tumors into low or high expression groups using boundary

Add this classification to the `colData(e)` object

```
top_nhrs_ensg <- top_nhrs$geneNm

for(i in 1:length(top_nhrs_ensg)){
  classific <- returnClassif(tum_matr = e, tum_res = res2, gene = top_nhrs$geneNm[i])
  colData(e) <- bindClassToColData(cdat = colData(e), classific)
}
```

## 3.2 Check for technical bias by sequencing plate for the 6 genes.

```
seqPlateClassif <- select(as.data.frame(colData(e)), starts_with("classif_")) %>%
  data.frame(seqPlate=colData(e)$seqPlate) %>%
  gather(key = "ensembl_gene_id", value = "bin_expr", 1:(ncol(.)-1))

seqPlateOrd <- orderSeqPlate(seqPlateClassif)
seqPlateCounts <- countSeqPlate(seqPlateClassif, seqPlateOrd$seqPlate)

numbRows <- 2
numbPanels <- length(unique(seqPlateCounts$ensembl_gene_id))
numbPerRow <- numbPanels / numbRows
seqPlateCounts_sbst1 <- subset(seqPlateCounts,as.character(ensembl_gene_id) %in% as.character(unique(seqPlateCounts$ensembl_gene_id)[1:numbPerRow]))
seqPlateCounts_sbst2 <- subset(seqPlateCounts,as.character(ensembl_gene_id) %in% as.character(unique(seqPlateCounts$ensembl_gene_id)[numbPerRow+ 1:numbPanels]))

ggplot(data=seqPlateCounts_sbst1, aes(x=seqPlate, y=n, fill=bin_expr)) + ggSeqPlate
```

```
ggplot(data=seqPlateCounts_sbst2, aes(x=seqPlate, y=n, fill=bin_expr)) + ggSeqPlate
```

```
#do a chi sq test between all expression plates and expression statuses
chiSqRes <- c()
for(var in unique(seqPlateCounts$ensembl_gene_id)){
  print(var)
  seqPlateCounts_sbst <- subset(seqPlateCounts, ensembl_gene_id %in% var)
  chiSqPval <- chisq.test(seqPlateCounts_sbst$bin_expr, seqPlateCounts_sbst$n)$p.value
  chiSqRes <- c(chiSqRes, chiSqPval)
}
```

```
## [1] "classif_ENSG00000082175"
## [1] "classif_ENSG00000091831"
## [1] "classif_ENSG00000123358"
## [1] "classif_ENSG00000132170"
## [1] "classif_ENSG00000151090"
## [1] "classif_ENSG00000169083"
```

```
names(chiSqRes) <- unique(seqPlateCounts$ensembl_gene_id) #levels(seqPlateCounts$ensembl_gene_id)
chiSqRes.adj <- p.adjust(chiSqRes, method="BH")
chiSqRes.adj
```

```
## classif_ENSG00000082175 classif_ENSG00000091831 classif_ENSG00000123358 
##               0.3151892               0.3151892               0.4942243 
## classif_ENSG00000132170 classif_ENSG00000151090 classif_ENSG00000169083 
##               0.1002790               0.1002790               0.4942243
```

## 3.3 Check for technical bias by sequencing plate for all genes.

```
#boundAll <- apply(assay(en), 1, function(x) twoSdBelowMean(mean(x), sd(x)))
#boundAll is res2$lowerBound
res2_arrange <- res2 %>% arrange(geneNm)
boundAll <- res2_arrange %>% pull(lowerBound) %>% setNames(., nm = res2_arrange$geneNm)

sum(res2$lowerBound > 0) #all genes with boundary greater than 0
```

```
## [1] 17034
```

```
#is only a function of the adjacent normal data
exprLessThanBound <- rowSums(assay(e) < boundAll)
exprGrThanBound <- rowSums(assay(e) > boundAll)

filtCutoffLower <- 0.2

boundFiltGenes <- boundAll[{exprGrThanBound/ncol(assay(e)) > filtCutoffLower} & {exprGrThanBound/ncol(assay(e)) < 1-filtCutoffLower} & boundAll > 0]

binIndxMatrGrTh2 <- ifelse(assay(e)[names(boundFiltGenes),] > boundFiltGenes, 1, 0)


seqPlateClassif_all <- binIndxMatrGrTh2 %>% 
  t() %>%
  data.frame(seqPlate=colData(e)$seqPlate) %>%
  gather(key = "ensembl_gene_id", value = "bin_expr", 1:(ncol(.)-1))

seqPlateOrdAll <- orderSeqPlate(seqPlateClassif_all)
seqPlateCountsAll <- countSeqPlate(seqPlateClassif_all, seqPlateOrdAll$seqPlate)

chiSqResAll <- c()
for(var in levels(seqPlateCountsAll$seqPlate)){
  seqPlateCounts_sbst <- subset(seqPlateCountsAll, seqPlate %in% var)
  chiSqPval <- chisq.test(seqPlateCounts_sbst$bin_expr,
                          seqPlateCounts_sbst$n)$p.value
  chiSqResAll <- c(chiSqResAll, chiSqPval)
}

names(chiSqResAll) <- levels(seqPlateCountsAll$ensembl_gene_id)
chiSqResAll.adj <- p.adjust(chiSqResAll, method="BH")
min(chiSqResAll.adj)
```

```
## [1] 0
```

# 4 Identify survival differences based on NHR expression

```
bound_nuHrRcpt_classif <- colData(e) %>% 
  as.data.frame() %>%
  select(starts_with("classif_"))
rownames(bound_nuHrRcpt_classif) <- rownames(colData(e))

abBlwDf2.nhr.top <- res2 %>% filter(is_nuclr_recpt) %>% head()

plot_surv_pval <- c()
geneNms <- c()
for(col in 1:length(colnames(bound_nuHrRcpt_classif))){
  ensg_id <- substr(x = colnames(bound_nuHrRcpt_classif)[col], 9, 23)
  gnNm <- abBlwDf2.nhr.top %>% 
    filter(ensg_id == geneNm) %>% 
    pull(hgnc_symbol)
  print(gnNm)
  print(col)
  receptLoss::plotReceptLoss(exprMatrNml=assay(en), exprMatrTum=assay(e), rldf = res2, geneName=ensg_id, clrs = set2[4:3], addToTitle = paste0(" - ", gnNm))
  
  ggsave(filename = here("output", paste0(gnNm, "_hist.png")))
  plot_surv <- plotSurvAndReturnStat(timeToEvent = colData(e)$days_last_fu_5yr/365.25, 
                                     alive0dead1 = colData(e)$vital_stat_5yr, 
                                     classif = colData(e)[,colnames(bound_nuHrRcpt_classif)[col]],
                                     surv.col = set2[1:2], gnNm = gnNm)
  ggsave(filename = here("output", paste0(gnNm, "_surv.png")))
  plot_surv_pval <- c(plot_surv_pval, pvalFxn(plot_surv))
  geneNms <- c(geneNms, gnNm)
}
```

```
## [1] "PGR"
## [1] 1
```

```
## Call:
## survdiff(formula = Surv(timeToEvent, alive0dead1) ~ classif)
## 
##             N Observed Expected (O-E)^2/E (O-E)^2/V
## classif=0 225       52     32.2     12.17      20.1
## classif=1 317       30     49.8      7.87      20.1
## 
##  Chisq= 20.1  on 1 degrees of freedom, p= 7e-06
```

```
## [1] "ESR1"
## [1] 2
```

```
## Call:
## survdiff(formula = Surv(timeToEvent, alive0dead1) ~ classif)
## 
##             N Observed Expected (O-E)^2/E (O-E)^2/V
## classif=0 123       33     16.8     15.76      19.8
## classif=1 419       49     65.2      4.05      19.8
## 
##  Chisq= 19.8  on 1 degrees of freedom, p= 8e-06
```

```
## [1] "PPARG"
## [1] 3
```

```
## Call:
## survdiff(formula = Surv(timeToEvent, alive0dead1) ~ classif)
## 
##             N Observed Expected (O-E)^2/E (O-E)^2/V
## classif=0 123       22     18.7     0.565     0.733
## classif=1 419       60     63.3     0.168     0.733
## 
##  Chisq= 0.7  on 1 degrees of freedom, p= 0.4
```

```
## [1] "AR"
## [1] 4
```

```
## Call:
## survdiff(formula = Surv(timeToEvent, alive0dead1) ~ classif)
## 
##             N Observed Expected (O-E)^2/E (O-E)^2/V
## classif=0 390       65     58.9     0.624      2.22
## classif=1 152       17     23.1     1.594      2.22
## 
##  Chisq= 2.2  on 1 degrees of freedom, p= 0.1
```

```
## [1] "NR4A1"
## [1] 5
```

```
## Call:
## survdiff(formula = Surv(timeToEvent, alive0dead1) ~ classif)
## 
##             N Observed Expected (O-E)^2/E (O-E)^2/V
## classif=0 167       27     24.9    0.1847     0.265
## classif=1 375       55     57.1    0.0803     0.265
## 
##  Chisq= 0.3  on 1 degrees of freedom, p= 0.6
```

```
## [1] "THRB"
## [1] 6
```

```
## Call:
## survdiff(formula = Surv(timeToEvent, alive0dead1) ~ classif)
## 
##             N Observed Expected (O-E)^2/E (O-E)^2/V
## classif=0 122        6     19.8      9.63      12.7
## classif=1 420       76     62.2      3.07      12.7
## 
##  Chisq= 12.7  on 1 degrees of freedom, p= 4e-04
```

```
plot_surv_pval.adj <- p.adjust(plot_surv_pval, method = "BH")
names(plot_surv_pval.adj) <- geneNms

#expression of THRb in normal and canceroustissue 
isof = "ENSG00000151090"
tum_class <- c(colData(e)[,"classif_ENSG00000151090"], rep(2,ncol(assay(en)))) %>%
  as_tibble(x = list(cls = .))

tidyDf <- as.data.frame(cbind(as.numeric(c(assay(e)[isof,], assay(en)[isof,])),
                      as.factor(c(rep("tumor",ncol(assay(e))),
                                  rep("normal",ncol(assay(en)))))))
tidyDf %>% bind_cols(tum_class) %>% group_by(cls) %>% summarise(mn = mean(V1))
```

```
## # A tibble: 3 x 2
##     cls    mn
##   <dbl> <dbl>
## 1     0 0.271
## 2     1 2.23 
## 3     2 3.44
```

```
#hist of expr of PGR in normal and cancer
plotGene_bothHist(exprNml=assay(en), exprTum=assay(e), isof = "ENSG00000082175", title="", clrs = set2[4:3])
```

```
## [1] 6.686996
## [1] 3.950465
```

```
plotGene_oneHist(exprNml=assay(en), exprTum=assay(e), isof = "ENSG00000082175", title="", clrs = set2[4:3], vert_line=FALSE)
```

```
## [1] 6.686996
## [1] 3.950465
```

```
plotGene_oneHistNmlCurve(exprNml=assay(en), exprTum=assay(e), isof = "ENSG00000082175", title="", clrs = set2[4:3], vert_line=FALSE)
```

```
## [1] 6.686996
## [1] 3.950465
```

```
plotGene_oneHistNmlCurve(exprNml=assay(en), exprTum=assay(e), isof = "ENSG00000082175", title="", clrs = set2[4:3], vert_line=TRUE)
```

```
## [1] 6.686996
## [1] 3.950465
```

```
plotGene_oneHistNmlCurve(exprNml=assay(en), exprTum=assay(e), isof = "ENSG00000082175", title="", clrs = set2[4:3], vert_line=FALSE)
```

```
## [1] 6.686996
## [1] 3.950465
```

```
plotGene_oneHistNmlCurve(exprNml=assay(en), exprTum=assay(e), isof = "ENSG00000082175", title="", clrs = set2[4:3], vert_line=FALSE, histogram = FALSE)
```

```
## [1] 6.686996
## [1] 3.950465
```

```
plotGeneHist2(exprNml=assay(en), exprTum=assay(e), isof = ensg_id, title=gnNm, clrs = set2[4:3])
```

```
## [1] 3.444236
## [1] 0.6102247
```

## 4.1 Identify clinical features of THRB-low subtype

For the following clinical features listed in `cols_of_interest`, there is a 2 x n table showing the distribution of features between tumors that lose THRB expression (0) and tumors that do not lose THRB expression (1). Then, there are 2 additional tables: one representing the proportion of all patients within a given group, and the other representing the proportion of patients normalized to the total # of patients within each THRB group.

For each feature, following each table, there is a visual representation of the last table, containing an unadjusted p-value obtained from the chi-sq test that compares THRB expression status (0 or 1) against the variable of interest (all variables tested happen to be categorical). A list of adjusted p-values across all tested variables appears at the end of this section. The levels of each variable are represented along the outer left y axis, and their representation among each group corresponds, in order, with the colors shown within each bar plot (the inner left y axis contains an incomplete list of variables and is a plotting artifact).

For example, for the variable “subtype\_IntegrativeCluster”, ‘CN high’ tumors represent ~5% of all tumors that lose THRB expression, while ‘CN high’ tumors represent ~15% of all tumors that do not lose THRB expression. The proportion of tumors represented is shown along the right y axis.

```
print_tbl <- function(thrb_column, var_column){
      print(table(thrb_column, var_column) %>% prop.table %>% t %>% data.frame() %>% spread(thrb_column, Freq) %>% map_df(rev) %>% mutate(lvl_sum = `0` + `1`) %>% adorn_totals("row"))
}

print_tbl_rel <- function(thrb_column, var_column){
      print(table(thrb_column, var_column) %>% prop.table(1) %>% t %>% data.frame() %>% spread(thrb_column, Freq) %>% map_df(rev) %>% adorn_totals("row") )
}

cols_of_interest <- c("subtype_IntegrativeCluster", "subtype_msi_status_7_marker_call", "subtype_tumor_grade", "subtype_histology", "subtype_X2009stagegroup") #"subtype_cna_cluster_k4", "subtype_mrna_expression_cluster",
pval_store <- rep(NA, length(cols_of_interest))
for(i in 1:length(cols_of_interest)){
  thrb_classif <- factor(colData(e)[,"classif_ENSG00000151090"])
  col_int <- factor(colData(e)[,cols_of_interest[i]])
  pval_store[i] <- chisq.test(thrb_classif, col_int)$p.value
  print("Absolute # of patients in each group")
  print(table(colData(e)[,"classif_ENSG00000151090"], colData(e)[,cols_of_interest[i]]))
  print("Proportion of patients in each group")
  print_tbl(colData(e)[,"classif_ENSG00000151090"], colData(e)[,cols_of_interest[i]])
  print("Proportion of patients within each THRB group (0 or 1)")
  print_tbl_rel(colData(e)[,"classif_ENSG00000151090"], colData(e)[,cols_of_interest[i]])
  plot(thrb_classif, 
       col_int, 
       xlab="THRB status (0=loss of expr)",
       ylab=paste0(levels(col_int),
                   collapse = ", "),
       
       main=paste0(cols_of_interest[i], "; unadj. pval = ", round(pval_store[i],5)), 
       cex.lab=2.5, cex.axis=2.5, cex.main=1.5, cex.sub=2.5, yaxt='n', xaxt='n', ann=FALSE)
}
```

```
## [1] "Absolute # of patients in each group"
##    
##     CN high CN low Missing MSI Notassigned POLE
##   0       6     16      33  28          34    5
##   1      54     74     139  37         104   12
## [1] "Proportion of patients in each group"
##   var_column           0          1    lvl_sum
##         POLE 0.009225092 0.02214022 0.03136531
##  Notassigned 0.062730627 0.19188192 0.25461255
##          MSI 0.051660517 0.06826568 0.11992620
##      Missing 0.060885609 0.25645756 0.31734317
##       CN low 0.029520295 0.13653137 0.16605166
##      CN high 0.011070111 0.09963100 0.11070111
##        Total 0.225092251 0.77490775 1.00000000
## [1] "Proportion of patients within each THRB group (0 or 1)"
##   var_column          0          1
##         POLE 0.04098361 0.02857143
##  Notassigned 0.27868852 0.24761905
##          MSI 0.22950820 0.08809524
##      Missing 0.27049180 0.33095238
##       CN low 0.13114754 0.17619048
##      CN high 0.04918033 0.12857143
##        Total 1.00000000 1.00000000
```

```
## [1] "Absolute # of patients in each group"
##    
##     Indeterminant Missing MSI-H MSI-L MSS
##   0             0      33    52     3  34
##   1             3     139    75    17 186
## [1] "Proportion of patients in each group"
##     var_column           0           1     lvl_sum
##            MSS 0.062730627 0.343173432 0.405904059
##          MSI-L 0.005535055 0.031365314 0.036900369
##          MSI-H 0.095940959 0.138376384 0.234317343
##        Missing 0.060885609 0.256457565 0.317343173
##  Indeterminant 0.000000000 0.005535055 0.005535055
##          Total 0.225092251 0.774907749 1.000000000
## [1] "Proportion of patients within each THRB group (0 or 1)"
##     var_column          0           1
##            MSS 0.27868852 0.442857143
##          MSI-L 0.02459016 0.040476190
##          MSI-H 0.42622951 0.178571429
##        Missing 0.27049180 0.330952381
##  Indeterminant 0.00000000 0.007142857
##          Total 1.00000000 1.000000000
```

```
## [1] "Absolute # of patients in each group"
##    
##     Grade 1 Grade 2 Grade 3 Missing
##   0      22      34      33      33
##   1      67      70     144     139
## [1] "Proportion of patients in each group"
##  var_column          0         1   lvl_sum
##     Missing 0.06088561 0.2564576 0.3173432
##     Grade 3 0.06088561 0.2656827 0.3265683
##     Grade 2 0.06273063 0.1291513 0.1918819
##     Grade 1 0.04059041 0.1236162 0.1642066
##       Total 0.22509225 0.7749077 1.0000000
## [1] "Proportion of patients within each THRB group (0 or 1)"
##  var_column         0         1
##     Missing 0.2704918 0.3309524
##     Grade 3 0.2704918 0.3428571
##     Grade 2 0.2786885 0.1666667
##     Grade 1 0.1803279 0.1595238
##       Total 1.0000000 1.0000000
```

```
## [1] "Absolute # of patients in each group"
##    
##     Endometrioid Missing Mixed Serous
##   0           82      33     1      6
##   1          222     139    12     47
## [1] "Proportion of patients in each group"
##    var_column           0          1    lvl_sum
##        Serous 0.011070111 0.08671587 0.09778598
##         Mixed 0.001845018 0.02214022 0.02398524
##       Missing 0.060885609 0.25645756 0.31734317
##  Endometrioid 0.151291513 0.40959410 0.56088561
##         Total 0.225092251 0.77490775 1.00000000
## [1] "Proportion of patients within each THRB group (0 or 1)"
##    var_column           0          1
##        Serous 0.049180328 0.11190476
##         Mixed 0.008196721 0.02857143
##       Missing 0.270491803 0.33095238
##  Endometrioid 0.672131148 0.52857143
##         Total 1.000000000 1.00000000
```

```
## [1] "Absolute # of patients in each group"
##    
##     Missing Stage I Stage II Stage III Stage IV Unknown
##   0      33      63        9        17        0       0
##   1     139     188       16        57       17       3
## [1] "Proportion of patients in each group"
##  var_column          0           1     lvl_sum
##     Unknown 0.00000000 0.005535055 0.005535055
##    Stage IV 0.00000000 0.031365314 0.031365314
##   Stage III 0.03136531 0.105166052 0.136531365
##    Stage II 0.01660517 0.029520295 0.046125461
##     Stage I 0.11623616 0.346863469 0.463099631
##     Missing 0.06088561 0.256457565 0.317343173
##       Total 0.22509225 0.774907749 1.000000000
## [1] "Proportion of patients within each THRB group (0 or 1)"
##  var_column          0           1
##     Unknown 0.00000000 0.007142857
##    Stage IV 0.00000000 0.040476190
##   Stage III 0.13934426 0.135714286
##    Stage II 0.07377049 0.038095238
##     Stage I 0.51639344 0.447619048
##     Missing 0.27049180 0.330952381
##       Total 1.00000000 1.000000000
```

```
p.adjust(pval_store, method = "BH") %>% setNames(cols_of_interest)
```

```
##       subtype_IntegrativeCluster subtype_msi_status_7_marker_call 
##                     4.937468e-04                     4.435998e-06 
##              subtype_tumor_grade                subtype_histology 
##                     3.600516e-02                     3.090518e-02 
##          subtype_X2009stagegroup 
##                     6.267951e-02
```

```
## post hoc test - thrb and msi and integrative
integrative_msi_1_other_0 <- colData(e)[,cols_of_interest[1]] %>% sapply(function(x) ifelse(x == "MSI", 0, 1))
pv_integ <- stats::fisher.test(thrb_classif, integrative_msi_1_other_0) #integrative
pv_integ
```

```
## 
##  Fisher's Exact Test for Count Data
## 
## data:  thrb_classif and integrative_msi_1_other_0
## p-value = 9.757e-05
## alternative hypothesis: true odds ratio is not equal to 1
## 95 percent confidence interval:
##  1.719876 5.460337
## sample estimates:
## odds ratio 
##   3.075246
```

```
msi_msih_0_other_1 <- colData(e)[,cols_of_interest[2]] %>% sapply(function(x) ifelse(x == "MSI-H", 0, 1)) #msi
pv_msi <- stats::fisher.test(thrb_classif, msi_msih_0_other_1) #integrative
pv_msi
```

```
## 
##  Fisher's Exact Test for Count Data
## 
## data:  thrb_classif and msi_msih_0_other_1
## p-value = 5.621e-08
## alternative hypothesis: true odds ratio is not equal to 1
## 95 percent confidence interval:
##  2.148969 5.404964
## sample estimates:
## odds ratio 
##     3.4079
```

```
p.adjust(c(pv_integ$p.value, pv_msi$p.value), method = "BH") %>% setNames(c("integ", "msi"))
```

```
##        integ          msi 
## 9.756512e-05 1.124207e-07
```

## 4.2 Look at survival differences between endometroid tumors

```
ensg_id <- "ENSG00000151090" #THRB
gnNm <- abBlwDf2.nhr.top %>% 
  filter(ensg_id == geneNm) %>% 
  pull(hgnc_symbol)

colData_e_endometroid <- colData(e) %>% as_tibble %>% filter(subtype_histology == "Endometrioid") %>%
  DataFrame()

plot_surv <- plotSurvAndReturnStat(timeToEvent = colData_e_endometroid$days_last_fu_5yr/365.25, 
                                   alive0dead1 = colData_e_endometroid$vital_stat_5yr, 
                                   classif = colData_e_endometroid[,colnames(bound_nuHrRcpt_classif)[col]],
                                   surv.col = set2[1:2], gnNm = gnNm)
```

```
## Call:
## survdiff(formula = Surv(timeToEvent, alive0dead1) ~ classif)
## 
##             N Observed Expected (O-E)^2/E (O-E)^2/V
## classif=0  82        6     9.47     1.270      1.78
## classif=1 222       27    23.53     0.511      1.78
## 
##  Chisq= 1.8  on 1 degrees of freedom, p= 0.2
```

```
ggsave(filename = here("output", paste0(gnNm, "_endometrioid_surv.png")))
plot_surv_pval <- c(plot_surv_pval, pvalFxn(plot_surv))
```

## 4.3 Look at survival differences between serous tumors

```
colData_e_serous <- colData(e) %>% as_tibble %>% filter(subtype_histology == "Serous") %>%
  DataFrame()

plot_surv <- plotSurvAndReturnStat(timeToEvent = colData_e_serous$days_last_fu_5yr/365.25, 
                                   alive0dead1 = colData_e_serous$vital_stat_5yr, 
                                   classif = colData_e_serous[,colnames(bound_nuHrRcpt_classif)[col]],
                                   surv.col = set2[1:2], gnNm = gnNm)
```

```
## Call:
## survdiff(formula = Surv(timeToEvent, alive0dead1) ~ classif)
## 
##            N Observed Expected (O-E)^2/E (O-E)^2/V
## classif=0  6        0     1.75     1.746      2.01
## classif=1 47       14    12.25     0.249      2.01
## 
##  Chisq= 2  on 1 degrees of freedom, p= 0.2
```

```
ggsave(filename = here("output", paste0(gnNm, "_serous_surv.png")))
plot_surv_pval <- c(plot_surv_pval, pvalFxn(plot_surv))
```

# 5 Examine combinatorial relationships between survival groups

```
colData(e)$classif_thrb_esr1_pgr <- apply(colData(e)[,c("classif_ENSG00000151090", "classif_ENSG00000091831","classif_ENSG00000082175")], 1, paste0, collapse = "") 

colData(e)$classif_esr1_pgr <- apply(colData(e)[,c( "classif_ENSG00000091831", "classif_ENSG00000082175")], 1, paste0, collapse = "") 

#must have at least 3 observations per group
subgrps_grth_3 <- colData(e)$classif_thrb_esr1_pgr %>% table %>% .[.>=3] %>% names
filt_vect <- colData(e)$classif_thrb_esr1_pgr %in% subgrps_grth_3 #c("000", "011", "111", "100")#subgrps_grth_3
plot_5combo <- plotSurvAndReturnStat(timeToEvent = {colData(e)$days_last_fu_5yr/365.25}[filt_vect], 
                            alive0dead1 = {colData(e)$vital_stat_5yr}[filt_vect], 
                            classif = {colData(e)$classif_thrb_esr1_pgr}[filt_vect],
                            surv.col = set2[c(6:4, 1:3)], gnNm = "")
```

```
## Call:
## survdiff(formula = Surv(timeToEvent, alive0dead1) ~ classif)
## 
##               N Observed Expected (O-E)^2/E (O-E)^2/V
## classif=000  18        1     2.55     0.943     0.974
## classif=010  21        1     3.49     1.773     1.853
## classif=011  83        4    13.58     6.759     8.128
## classif=100 103       31    13.89    21.066    25.471
## classif=110  83       19    11.93     4.190     4.916
## classif=111 232       25    35.56     3.136     5.592
## 
##  Chisq= 38  on 5 degrees of freedom, p= 4e-07
```

```
#now, just look at ER/PGR negative depending on THRB status
filt_vect2 <- colData(e)$classif_thrb_esr1_pgr %in% c("000", "100")#subgrps_grth_3
filt_vect2 <- colData(e)$classif_thrb_esr1_pgr %in% c("011", "111")#subgrps_grth_3
filt_vect2 <- colData(e)$classif_thrb_esr1_pgr %in% c("100", "111")#subgrps_grth_3 #THRB pos
filt_vect2 <- colData(e)$classif_thrb_esr1_pgr %in% c("000", "011")#subgrps_grth_3 #THRB neg

plot_5combo <- plotSurvAndReturnStat(timeToEvent = {colData(e)$days_last_fu_5yr/365.25}[filt_vect2], 
                            alive0dead1 = {colData(e)$vital_stat_5yr}[filt_vect2], 
                            classif = {colData(e)$classif_thrb_esr1_pgr}[filt_vect2],
                            surv.col = set2[1:2], gnNm = "")
```

```
## Call:
## survdiff(formula = Surv(timeToEvent, alive0dead1) ~ classif)
## 
##              N Observed Expected (O-E)^2/E (O-E)^2/V
## classif=000 18        1    0.821   0.03882    0.0465
## classif=011 83        4    4.179   0.00763    0.0465
## 
##  Chisq= 0  on 1 degrees of freedom, p= 0.8
```

```
subgrps_esr_pgr <- colData(e)$classif_esr1_pgr %>% table %>% .[.>=3] %>% names
filt_vect3 <- colData(e)$classif_esr1_pgr %in% subgrps_esr_pgr#c("000", "011") 

plot_esr_pgr <- plotSurvAndReturnStat(timeToEvent = {colData(e)$days_last_fu_5yr/365.25}[filt_vect3], 
                            alive0dead1 = {colData(e)$vital_stat_5yr}[filt_vect3], 
                            classif = {colData(e)$classif_esr1_pgr}[filt_vect3],
                            surv.col = set2[1:3], gnNm = "")
```

```
## Call:
## survdiff(formula = Surv(timeToEvent, alive0dead1) ~ classif)
## 
##              N Observed Expected (O-E)^2/E (O-E)^2/V
## classif=00 121       32     16.4     14.72     18.50
## classif=10 104       20     15.4      1.36      1.68
## classif=11 315       29     49.1      8.25     21.02
## 
##  Chisq= 24.4  on 2 degrees of freedom, p= 5e-06
```

```
#double positive and double negative
subgrps_esr_pgr_dp_dn <- c("00", "11")#colData(e)$classif_esr1_pgr %>% table %>% .[.>=3] %>% names
filt_vect4 <- colData(e)$classif_esr1_pgr %in% subgrps_esr_pgr_dp_dn#c("000", "011") 

plot_esr_pgr_dp_dn <- plotSurvAndReturnStat2(timeToEvent = {colData(e)$days_last_fu_5yr/365.25}[filt_vect4], 
                            alive0dead1 = {colData(e)$vital_stat_5yr}[filt_vect4], 
                            classif = {colData(e)$classif_esr1_pgr}[filt_vect4],
                            surv.col = set2[1:2], gnNm = "")
```

```
## Call:
## survdiff(formula = Surv(timeToEvent, alive0dead1) ~ classif)
## 
##              N Observed Expected (O-E)^2/E (O-E)^2/V
## classif=00 121       32     15.2     18.62      24.9
## classif=11 315       29     45.8      6.17      24.9
## 
##  Chisq= 24.9  on 1 degrees of freedom, p= 6e-07
```

```
pv_00_11 <- pvalFxn(plot_esr_pgr_dp_dn$fit.stat)
pv_00_11
```

```
## [1] 6.174277e-07
```

```
#plot_esr_pgr_dp_dn

#risk of death at 5 years in each group
sum_survfit_dp_dn <- summary(plot_esr_pgr_dp_dn$fit, times = 5)
sum_survfit_dp_dn$surv #risk of death at 5 years in each group
```

```
## [1] 0.6064199 0.8384406
```

```
diff(sum_survfit_dp_dn$surv) #difference in 5 year risk of death btw groups
```

```
## [1] 0.2320207
```

```
ggsave(filename = here::here("output/00_vs_11.png"), device = "png", width = 5)


#plot 000 vs 100
filt_vect5 <- colData(e)$classif_thrb_esr1_pgr %in% c("000", "100") 

# plotSurvAndReturnStat2 does the same as plotSurvAndReturnStat but just returns an additional survfit object ('fit') in a list
plot_thrb_pos_vs_neg_esr_pgr_dbl_neg <- plotSurvAndReturnStat2(timeToEvent = {colData(e)$days_last_fu_5yr/365.25}[filt_vect5], 
                            alive0dead1 = {colData(e)$vital_stat_5yr}[filt_vect5], 
                            classif = {colData(e)$classif_thrb_esr1_pgr}[filt_vect5],
                            surv.col = set2[4:5], gnNm = "")
```

```
## Call:
## survdiff(formula = Surv(timeToEvent, alive0dead1) ~ classif)
## 
##               N Observed Expected (O-E)^2/E (O-E)^2/V
## classif=000  18        1     4.89     3.094      3.66
## classif=100 103       31    27.11     0.558      3.66
## 
##  Chisq= 3.7  on 1 degrees of freedom, p= 0.06
```

```
plot_thrb_pos_vs_neg_esr_pgr_dbl_neg
```

```
## $fit.stat
## Call:
## survdiff(formula = Surv(timeToEvent, alive0dead1) ~ classif)
## 
##               N Observed Expected (O-E)^2/E (O-E)^2/V
## classif=000  18        1     4.89     3.094      3.66
## classif=100 103       31    27.11     0.558      3.66
## 
##  Chisq= 3.7  on 1 degrees of freedom, p= 0.06 
## 
## $fit
## Call: survfit(formula = Surv(timeToEvent, alive0dead1) ~ classif)
## 
##               n events median 0.95LCL 0.95UCL
## classif=000  18      1     NA      NA      NA
## classif=100 103     31     NA    4.06      NA
```

```
pv_000_100 <- pvalFxn(plot_thrb_pos_vs_neg_esr_pgr_dbl_neg$fit.stat)
pv_000_100
```

```
## [1] 0.05568885
```

```
p.adjust(c(pv_00_11, pv_000_100), method = "BH")
```

```
## [1] 1.234855e-06 5.568885e-02
```

```
#percent alive at 5 years in each group
surv_tbl <- table({colData(e)$vital_stat_5yr}[filt_vect5], {colData(e)$classif_thrb_esr1_pgr}[filt_vect5]) %>% addmargins()
surv_perc_5yr <- surv_tbl[1,] / surv_tbl["Sum",]
surv_perc_5yr
```

```
##       000       100       Sum 
## 0.9444444 0.6990291 0.7355372
```

```
#risk of death at 5 years in each group
sum_survfit <- summary(plot_thrb_pos_vs_neg_esr_pgr_dbl_neg$fit, times = 5)
sum_survfit$surv #risk of death at 5 years in each group
```

```
## [1] 0.9375000 0.5584414
```

```
diff(sum_survfit$surv) #difference in 5 year risk of death btw groups
```

```
## [1] -0.3790586
```

```
ggsave(filename = here::here("output/000_vs_100.png"), device = "png", width = 5)

#plot 011 vs 111

filt_vect6 <- colData(e)$classif_thrb_esr1_pgr %in% c("011", "111") 

plot_thrb_pos_vs_neg_esr_pgr_dbl_neg <- plotSurvAndReturnStat(timeToEvent = {colData(e)$days_last_fu_5yr/365.25}[filt_vect6], 
                            alive0dead1 = {colData(e)$vital_stat_5yr}[filt_vect6], 
                            classif = {colData(e)$classif_thrb_esr1_pgr}[filt_vect6],
                            surv.col = set2[1:2], gnNm = "")
```

```
## Call:
## survdiff(formula = Surv(timeToEvent, alive0dead1) ~ classif)
## 
##               N Observed Expected (O-E)^2/E (O-E)^2/V
## classif=011  83        4     8.16     2.119      2.95
## classif=111 232       25    20.84     0.829      2.95
## 
##  Chisq= 3  on 1 degrees of freedom, p= 0.09
```

```
#plot 100 vs 111

filt_vect7 <- colData(e)$classif_thrb_esr1_pgr %in% c("100", "111") 

plot_thrb_pos_vs_neg_esr_pgr_dbl_neg <- plotSurvAndReturnStat(timeToEvent = {colData(e)$days_last_fu_5yr/365.25}[filt_vect7], 
                            alive0dead1 = {colData(e)$vital_stat_5yr}[filt_vect7], 
                            classif = {colData(e)$classif_thrb_esr1_pgr}[filt_vect7],
                            surv.col = set2[1:2], gnNm = "")
```

```
## Call:
## survdiff(formula = Surv(timeToEvent, alive0dead1) ~ classif)
## 
##               N Observed Expected (O-E)^2/E (O-E)^2/V
## classif=100 103       31     15.6     15.29      21.2
## classif=111 232       25     40.4      5.89      21.2
## 
##  Chisq= 21.2  on 1 degrees of freedom, p= 4e-06
```

```
ggsave(filename = here::here("output/100_vs_111.png"), device = "png", width = 5)
```

# 6 Test the association between NHR expression and mutations

Read in a list of established oncogenes and tumor suppressors from COSMIC V83, downloaded Dec. 2, 2017. Then, select the high impact mutations as called by mutect.

```
cosDat <- read.csv(file= here("data", "cosmic_mut", "Census_all_2017_12_2.csv"), header=T)

#run these two lines if readRDS does not exist
#maf_mut <- GDCquery_Maf("UCEC", pipelines = "mutect")#2018-12-09
#saveRDS(maf_mut, file = here("data", "TCGA-mut-mutect-ucec-2018-12.rds"))

mut <- readRDS(here("data","TCGA-mut-mutect-ucec-2018-12.rds"))
mut.hiImpct <- mut[mut$IMPACT %in% c("HIGH") & mut$Entrez_Gene_Id %in% cosDat$Entrez.GeneId,]
top50mutGenes <- sort(names(head(sort(table(mut.hiImpct$Hugo_Symbol),decreasing = T), 25)))
mut.hiImpct.top50 <- mut.hiImpct[mut.hiImpct$Hugo_Symbol %in% top50mutGenes,]
mut.hiImpct.top50.ptid <- names(table(mut.hiImpct.top50$Tumor_Sample_Barcode))

#which patients are associated with which gene mutations
#this is a list of patient TCGA ids
ptIdGeneMut <- sapply(top50mutGenes, function(x) unname(mut.hiImpct.top50[mut.hiImpct.top50$Hugo_Symbol %in% x,"Tumor_Sample_Barcode"]))

ptIdGeneMut.l <- lapply(ptIdGeneMut, function(x) mut.hiImpct.top50.ptid %in% x )
ptIdGeneMut.l.df <- do.call(rbind, ptIdGeneMut.l)
colnames(ptIdGeneMut.l.df) <- mut.hiImpct.top50.ptid
ptIdGeneMut.l.df <- ifelse(ptIdGeneMut.l.df, 1, 0)

#convert to summarized expt object
mutDat <- SummarizedExperiment(assays=list(mutation = as.matrix(ptIdGeneMut.l.df)))

ptInCommon <- intersect(substr(rownames(bound_nuHrRcpt_classif), 1,12), substr(colnames(assay(mutDat)), 1,12))

#create a histogram of mutation frequencies
mutFreq <- data.frame(freq=sort(rowSums(assay(mutDat[,sapply(ptInCommon, function(x) grep(x, colnames(mutDat)))])),decreasing = T))
mutFreq$gene <- rownames(mutFreq) 
mutFreq$gene <- factor(mutFreq$gene, levels=mutFreq$gene)

mf <- ggplot(mutFreq, aes(x=gene, y=freq/length(ptInCommon))) + 
  geom_bar(stat = "identity") + 
  ggBar + 
  ylab(paste0("Observed Frequency (N=", length(ptInCommon), ")"))

mf
```

## 6.1 Cluster hormone receptor and mutations status

Note: Both hormone receptor expression status and mutation status are binary variables.

```
#hormone receptor expression status
hrmnStatus.c <- clusterAndReorder(t(bound_nuHrRcpt_classif)[,substr(rownames(bound_nuHrRcpt_classif), 1,12) %in% ptInCommon], dimen = "both")
colnames(hrmnStatus.c) <- substr(colnames(hrmnStatus.c), 1,12)

#mutation status
mutDat.c <- clusterAndReorder(assay(mutDat)[,substr(colnames(assay(mutDat)), 1,12) %in% ptInCommon], dimen="both")
colnames(mutDat.c) <- substr(colnames(mutDat.c), 1,12)
```

## 6.2 Statistical testing (Fisher’s exact) between NHR expression and mutation

Are there associations between oncogene/tumor suppressor mutations and expression of NHRs? Use fisher’s exact test to look at relationship between each NHR and each mutation (50x6 matrix). Our 2 matrices are mutDat.c and hrmnStatus.c.sub.

```
hrmnStatus.c.sub <- hrmnStatus.c[,colnames(mutDat.c)]
rownames(hrmnStatus.c.sub) <- rownames(hrmnStatus.c.sub) %>%
                              substr(start = 9, stop = nchar(.)) %>%
                              match(abBlwDf2.nhr.top$geneNm) %>%
                              abBlwDf2.nhr.top[.,"hgnc_symbol"] %>% pull

orDat <- array(data = 0, dim = c(nrow(mutDat.c), nrow(hrmnStatus.c.sub), 2), dimnames = list(muts=rownames(mutDat.c), expr=rownames(hrmnStatus.c.sub), oddsRat=c("pValue", "OR")))

for(i in 1:nrow(mutDat.c)){
  for(j in 1:nrow(hrmnStatus.c.sub)){
    d1 <- c(mutDat.c[i,]) 
    d2 <- c(hrmnStatus.c.sub[j,])
    res <- fisher.test(x = d1, d2)
    orDat[i,j,1] <- res$p.value
    orDat[i,j,2] <- res$estimate
   }
}

p_val_adj <- orDat[,,1] %>% 
  as.vector %>% 
  p.adjust(method = "BH") %>% 
  matrix(ncol = ncol(orDat[,,1])) %>%
  as_tibble %>%
  purrr::set_names(colnames(orDat[,,1])) %>% 
  mutate(mut = rownames(orDat[,,1])) %>% 
  gather(key = "nhr", value = "val", 1:(ncol(.)-1)) %>%
  mutate(qval_or = "qval", sigStar = sig2star(val))
  
odd_rat <- orDat[,,2] %>% 
  data.frame() %>% 
  rownames_to_column("mut") %>% 
  gather(key = "nhr", value = "val", 2:ncol(.)) %>% 
  mutate(qval_or = "OR") %>% 
  as_tibble() %>%
  mutate(sigStar = "") %>%
  bind_rows(p_val_adj) %>%
  mutate(nhr = factor(nhr, levels=abBlwDf2.nhr.top$hgnc_symbol),
         mut = factor(mut, levels=rev(mutFreq$gene)))

odd_rat %>% filter(qval_or == "qval" & val < 0.05 & nhr == "THRB")
```

```
## # A tibble: 2 x 5
##   mut   nhr       val qval_or sigStar
##   <fct> <fct>   <dbl> <chr>   <chr>  
## 1 RNF43 THRB  0.00147 qval    *      
## 2 NSD1  THRB  0.00220 qval    *
```

```
qv_signif <- odd_rat %>% filter(qval_or == "qval" & val < 0.05)
or_qv_signif <- odd_rat %>% filter(paste0(mut, nhr) %in% paste0(qv_signif$mut, qv_signif$nhr))


md <- ggplot(subset(odd_rat,qval_or=="OR"), aes(y=mut, x=nhr)) +
  geom_tile(aes(fill=log(val,10)), color="black") + 
  theme_classic() + 
  theme(text = element_text(size=20, face="italic"),
        axis.ticks=element_blank(),
        axis.title=element_blank(),
        axis.line = element_blank(), 
        axis.text.x = element_text(angle=90, hjust = 1, vjust=0.5)) +
  scale_fill_gradient2(name="log10(Odds Ratio)") +
  geom_text(data=subset(odd_rat,qval_or=="qval"), aes(label=sigStar), size=6, nudge_y = -.2) +
  coord_fixed(ratio = .65)
md
```

```
mf
```

```
#md + mf + coord_flip() + scale_y_continuous(position = "right") + scale_x_discrete(limits = rev(levels(mutFreq$gene)))
```

## 6.3 Calculate Odds Ratio between each *pair* of genes

What’s the likelihood that when one is expressed, the other gene is also expressed? Use a cutoff of q = 0.01 (Fisher’s Exact) for pairwise relationship. Is THRB associated with any other expression statuses?

```
z <- bound_nuHrRcpt_classif
or_matrix <- matrix(ncol=ncol(z), nrow = ncol(z))
or_pval_matrix <- matrix(ncol=ncol(z), nrow = ncol(z))

for(i in 1:ncol(z)){
  for(j in 1:ncol(z)){
    if(i>=j) next
    xtab <- table(z[,i],z[,j])
    fisherTestRes <- fisher.test(xtab)
    
    oddsRat <- fisherTestRes$estimate
    or_matrix[j,i] <- oddsRat
    
    oddsRat_pval <- fisherTestRes$p.value
    or_pval_matrix[j,i] <- oddsRat_pval
  }
}

colnames(z) -> rownames(or_matrix) -> colnames(or_matrix) -> 
  rownames(or_pval_matrix) -> colnames(or_pval_matrix)

or_pval_matrix.m <- or_pval_matrix %>% 
    data.frame() %>%
  rownames_to_column("row") %>%
  gather(key = "variable", value = "pvalue", 2:ncol(.)) %>%
  filter(!is.na(pvalue)) %>%
  mutate(adjpvalue = p.adjust(pvalue, method="BH"),
         stars = cut(adjpvalue, 
                     breaks=c(-Inf, 0.0001, 0.001, 0.01, Inf), 
                     label=c("***", "**", "*", ""))) %>%
  mutate(row_var = paste0(row,":",variable)) %>%
  dplyr::select(-row, -variable)

or_matrix.m <- or_matrix %>%
  data.frame() %>%
  rownames_to_column("row") %>%
  gather(key = "variable", value = "OR", 2:ncol(.)) %>%
  filter(!is.na(OR)) %>%
  mutate(OR_cut = cut(OR ,breaks=c(0, 0.01, 0.1, 0.5, 2, 5, 50, Inf),
                      include.lowest=TRUE,
                      label=c("(0,0.01)","(0.01,0.1)", "(0.1,0.5)", 
                              "(0.5,2)", "(2,5)", "(5,50)", "(50,Inf)"))) %>%
  mutate(row_var = paste0(row,":",variable)) %>%
  dplyr::select(-row, -variable) %>% 
  left_join(or_pval_matrix.m, by="row_var") %>%
  separate(row_var, into = c("col1", "col2"), sep = ":")

gene_key <- data.frame(geneNms)
rownames(gene_key) <- colnames(bound_nuHrRcpt_classif)

or_matrix.m2 <- or_matrix.m %>% 
  mutate(col1 = factor(gene_key[col1,], levels=gene_key$geneNms),
         col2=factor(gene_key[col2,], levels=gene_key$geneNms))

colors_orr_corr_matr <- append(brewer.pal(11,"PuOr")[c(2,4,5,7,8,10)], "#ffffff", after=3) 

fig3a2 <- ggplot(or_matrix.m2, aes(col1, col2)) + 
  geom_tile(aes(fill=OR_cut),colour="black", size=1.5) + 
  geom_text(aes(label=stars), color="black", size=8, vjust=1) + 
  theme_classic() + 
  theme(axis.line = element_blank(), 
        text = element_text(size=20), 
        axis.text =element_text(size=16, color="black", face="italic"), 
        axis.text.x = element_text(angle = 90, hjust = 1, vjust=0.5),
        axis.ticks = element_blank(), 
        legend.key.size = unit(1.25,"cm")) + 
  scale_fill_manual(values = rev(colors_orr_corr_matr), 
                    name="Odds Ratio", drop=FALSE) + 
  coord_fixed(ratio = 1) + 
  scale_y_discrete(position="right") + 
  xlab("") + ylab("")

fig3a2
```

```
or_matrix.m2
```

```
##             OR   OR_cut  col1  col2       pvalue    adjpvalue stars
## 1  182.5051063 (50,Inf)  ESR1   PGR 1.614691e-54 2.422036e-53   ***
## 2    2.7964716    (2,5) PPARG   PGR 8.422597e-07 3.158474e-06   ***
## 3    4.5082786    (2,5)    AR   PGR 2.735161e-12 2.051371e-11   ***
## 4    1.1800049  (0.5,2) NR4A1   PGR 3.963425e-01 4.573183e-01      
## 5    0.5917032  (0.5,2)  THRB   PGR 1.637493e-02 3.508915e-02      
## 6    1.4155304  (0.5,2) PPARG  ESR1 1.429150e-01 1.948841e-01      
## 7    6.5455872   (5,50)    AR  ESR1 3.067757e-10 1.533878e-09   ***
## 8    1.9335731  (0.5,2) NR4A1  ESR1 2.597430e-03 7.792290e-03     *
## 9    0.5197975  (0.5,2)  THRB  ESR1 1.931341e-02 3.621264e-02      
## 10   1.4252549  (0.5,2)    AR PPARG 1.704500e-01 2.130625e-01      
## 11   1.6119866  (0.5,2) NR4A1 PPARG 2.679424e-02 4.041021e-02      
## 12   1.6887086  (0.5,2)  THRB PPARG 2.694014e-02 4.041021e-02      
## 13   1.0822868  (0.5,2) NR4A1    AR 7.564459e-01 7.564459e-01      
## 14   2.0351595    (2,5)  THRB    AR 5.801476e-03 1.450369e-02      
## 15   0.8340149  (0.5,2)  THRB NR4A1 4.383833e-01 4.696964e-01
```

# 7 Differential microRNA expression analysis

We look at the association between miRNAs and THRB expression

## 7.1 Volcano plot showing miRNA expression

```
bound_nuHrRcpt_classif_mirna_filt <- bound_nuHrRcpt_classif %>% .[rownames(.) %>% substr(1,12) %in% substr(colnames(mir_t), 1,12),]


mir_t2 <- rownames(bound_nuHrRcpt_classif_mirna_filt) %>% 
  substr(1,12) %>%
  sapply(grep, colnames(mir_t)) %>%
  unlist %>%
  unique %>%
  mir_t[,.] %>%
  mutate_all(.funs = ~log(.+1, 2))

mir_n2 <- colnames(en) %>% 
  substr(1,12) %>%
  sapply(grep, colnames(mir_n)) %>%
  unlist %>%
  mir_n[,.] %>%
  mutate_all(.funs = ~log(.+1, 2))

e_sub <- substr(colnames(mir_t2), 1,12) %>% 
  sapply(grep,colnames(e)) %>%
  e[,.]
en_sub <- substr(colnames(mir_n2), 1,12) %>% 
  sapply(grep,colnames(en)) %>%
  en[,.]


#############
i=6
classif <- colnames(bound_nuHrRcpt_classif)[i]#"classif_ENSG00000082175"
vars <- c(classif, "seqPlate", "hosp")
colData_e_stdze <- colData(e_sub)[,vars]
dsgn <- model.matrix(~ colData(e_sub)[,classif] + colData(e_sub)[,"seqPlate"] + colData(e_sub)$hosp)
allDeGenes <- returnDeMirnaLimma(mir_t2, dsgn, colOfInt = 2, retAll=T)

logFCThr = 1
adjPvalThresh = 0.001

allDeGenes2 <- allDeGenes %>% rownames() %>% 
  as.numeric() %>% mir$miRNA_ID[.] %>%
  cbind(allDeGenes, mirna=.) %>%
  as_tibble() %>%
  mutate(mirna = as.character(mirna),
         mirna_label = ifelse(abs(logFC) > logFCThr & adj.P.Val < adjPvalThresh,
                              mirna,""))

rowIdxDeMirna <- rownames(allDeGenes)[1] %>% as.numeric
mir$miRNA_ID[rowIdxDeMirna]
```

```
## [1] "hsa-mir-146a"
```

```
#make a volcano plot
ggplot(allDeGenes2, 
       aes(x = logFC, y = -log(adj.P.Val, 10))) +
  theme_bw() + geom_vline(xintercept = c(-logFCThr,logFCThr), 
                          linetype="dashed", color = "grey") + 
  geom_hline(yintercept = -log(adjPvalThresh,10), 
             linetype="dashed", color = "grey") +
  geom_point(alpha=0.5)+ 
  geom_text_repel(aes(label=mirna_label))+#aes(color=!inNeither & numHits > 3)) + 
  theme_classic() +
  theme(text  = element_text(size=18), legend.title = element_blank()) + 
  labs(size = 14, x = expression(paste(log[2], "(miRNA in THRB_Hi/THRB_Low)")), 
       y = (expression(-log[10](q~value)))) + 
  #annotate("segment", x=-0.7, y=0, xend=-1.15, yend=1.9, size=2, arrow=arrow(length = unit(0.03, "npc"), type="closed")) + 
  annotate("segment", x=-1.3, y=2.6, xend=-1.2, yend=3.5, size=1) +#, arrow=arrow(length = unit(0.03, "npc"), type="closed")) + 
  
  #annotate("text", x=-1.5, y=1, label="Upregulated in\nendomet. tumors\nwith low THRB expr.", hjust = 0) + 
  annotate("rect", xmin=-Inf, xmax=-1, ymin=3, ymax=Inf, alpha=0.2, fill="red") +
  annotate("text", x=-1.5, y=2, label="miRNAs upregulated\nin endomet. tumors\nwith low THRB expr.", hjust = 0)
```

```
#add arrow
```

## 7.2 Volcano plot showing miRNA differential expression in tumors with endometrioid histology

```
colData(e)$subtype_histology #will do differential expression for the endometrioid and serous tumors
```

```
##   [1] "Endometrioid" "Missing"      "Missing"      "Missing"      "Missing"     
##   [6] "Endometrioid" "Missing"      "Endometrioid" "Endometrioid" "Serous"      
##  [11] "Endometrioid" "Endometrioid" "Serous"       "Endometrioid" "Missing"     
##  [16] "Endometrioid" "Endometrioid" "Endometrioid" "Endometrioid" "Missing"     
##  [21] "Serous"       "Endometrioid" "Endometrioid" "Endometrioid" "Missing"     
##  [26] "Serous"       "Missing"      "Mixed"        "Missing"      "Missing"     
##  [31] "Missing"      "Endometrioid" "Endometrioid" "Endometrioid" "Endometrioid"
##  [36] "Endometrioid" "Serous"       "Endometrioid" "Endometrioid" "Serous"      
##  [41] "Missing"      "Mixed"        "Missing"      "Endometrioid" "Missing"     
##  [46] "Serous"       "Endometrioid" "Endometrioid" "Missing"      "Endometrioid"
##  [51] "Endometrioid" "Endometrioid" "Endometrioid" "Mixed"        "Missing"     
##  [56] "Endometrioid" "Endometrioid" "Missing"      "Endometrioid" "Endometrioid"
##  [61] "Endometrioid" "Missing"      "Endometrioid" "Missing"      "Mixed"       
##  [66] "Endometrioid" "Serous"       "Endometrioid" "Endometrioid" "Mixed"       
##  [71] "Endometrioid" "Endometrioid" "Endometrioid" "Endometrioid" "Endometrioid"
##  [76] "Missing"      "Serous"       "Serous"       "Endometrioid" "Endometrioid"
##  [81] "Missing"      "Missing"      "Missing"      "Endometrioid" "Serous"      
##  [86] "Endometrioid" "Serous"       "Missing"      "Endometrioid" "Endometrioid"
##  [91] "Missing"      "Missing"      "Endometrioid" "Serous"       "Endometrioid"
##  [96] "Endometrioid" "Missing"      "Endometrioid" "Missing"      "Serous"      
## [101] "Missing"      "Endometrioid" "Endometrioid" "Missing"      "Serous"      
## [106] "Endometrioid" "Endometrioid" "Missing"      "Endometrioid" "Endometrioid"
## [111] "Missing"      "Endometrioid" "Endometrioid" "Endometrioid" "Endometrioid"
## [116] "Endometrioid" "Endometrioid" "Endometrioid" "Missing"      "Endometrioid"
## [121] "Endometrioid" "Missing"      "Missing"      "Missing"      "Missing"     
## [126] "Endometrioid" "Missing"      "Missing"      "Missing"      "Serous"      
## [131] "Endometrioid" "Missing"      "Endometrioid" "Serous"       "Endometrioid"
## [136] "Endometrioid" "Endometrioid" "Endometrioid" "Missing"      "Endometrioid"
## [141] "Missing"      "Endometrioid" "Missing"      "Endometrioid" "Serous"      
## [146] "Missing"      "Endometrioid" "Endometrioid" "Endometrioid" "Endometrioid"
## [151] "Serous"       "Endometrioid" "Endometrioid" "Serous"       "Missing"     
## [156] "Endometrioid" "Endometrioid" "Endometrioid" "Endometrioid" "Endometrioid"
## [161] "Endometrioid" "Endometrioid" "Missing"      "Missing"      "Serous"      
## [166] "Missing"      "Missing"      "Missing"      "Serous"       "Endometrioid"
## [171] "Serous"       "Endometrioid" "Missing"      "Endometrioid" "Endometrioid"
## [176] "Missing"      "Missing"      "Endometrioid" "Endometrioid" "Missing"     
## [181] "Serous"       "Endometrioid" "Endometrioid" "Endometrioid" "Endometrioid"
## [186] "Endometrioid" "Serous"       "Endometrioid" "Endometrioid" "Endometrioid"
## [191] "Endometrioid" "Endometrioid" "Missing"      "Endometrioid" "Missing"     
## [196] "Endometrioid" "Serous"       "Endometrioid" "Endometrioid" "Endometrioid"
## [201] "Endometrioid" "Endometrioid" "Endometrioid" "Endometrioid" "Endometrioid"
## [206] "Missing"      "Endometrioid" "Missing"      "Endometrioid" "Endometrioid"
## [211] "Endometrioid" "Endometrioid" "Serous"       "Endometrioid" "Endometrioid"
## [216] "Endometrioid" "Endometrioid" "Endometrioid" "Missing"      "Endometrioid"
## [221] "Endometrioid" "Endometrioid" "Missing"      "Missing"      "Endometrioid"
## [226] "Missing"      "Serous"       "Endometrioid" "Missing"      "Endometrioid"
## [231] "Endometrioid" "Missing"      "Endometrioid" "Endometrioid" "Endometrioid"
## [236] "Missing"      "Missing"      "Endometrioid" "Endometrioid" "Missing"     
## [241] "Missing"      "Endometrioid" "Missing"      "Endometrioid" "Endometrioid"
## [246] "Missing"      "Missing"      "Endometrioid" "Missing"      "Endometrioid"
## [251] "Endometrioid" "Endometrioid" "Serous"       "Missing"      "Serous"      
## [256] "Endometrioid" "Endometrioid" "Missing"      "Endometrioid" "Missing"     
## [261] "Missing"      "Serous"       "Endometrioid" "Missing"      "Endometrioid"
## [266] "Endometrioid" "Endometrioid" "Serous"       "Missing"      "Endometrioid"
## [271] "Endometrioid" "Endometrioid" "Endometrioid" "Endometrioid" "Endometrioid"
## [276] "Missing"      "Endometrioid" "Endometrioid" "Serous"       "Endometrioid"
## [281] "Missing"      "Missing"      "Endometrioid" "Endometrioid" "Missing"     
## [286] "Endometrioid" "Missing"      "Serous"       "Endometrioid" "Endometrioid"
## [291] "Endometrioid" "Endometrioid" "Endometrioid" "Endometrioid" "Missing"     
## [296] "Endometrioid" "Missing"      "Endometrioid" "Endometrioid" "Endometrioid"
## [301] "Endometrioid" "Missing"      "Endometrioid" "Endometrioid" "Endometrioid"
## [306] "Missing"      "Missing"      "Missing"      "Endometrioid" "Endometrioid"
## [311] "Missing"      "Endometrioid" "Missing"      "Endometrioid" "Endometrioid"
## [316] "Missing"      "Missing"      "Serous"       "Endometrioid" "Endometrioid"
## [321] "Mixed"        "Missing"      "Serous"       "Endometrioid" "Endometrioid"
## [326] "Missing"      "Endometrioid" "Missing"      "Serous"       "Endometrioid"
## [331] "Serous"       "Missing"      "Missing"      "Endometrioid" "Endometrioid"
## [336] "Missing"      "Missing"      "Endometrioid" "Endometrioid" "Endometrioid"
## [341] "Endometrioid" "Serous"       "Endometrioid" "Endometrioid" "Missing"     
## [346] "Missing"      "Endometrioid" "Endometrioid" "Missing"      "Missing"     
## [351] "Missing"      "Missing"      "Endometrioid" "Missing"      "Endometrioid"
## [356] "Serous"       "Endometrioid" "Missing"      "Endometrioid" "Endometrioid"
## [361] "Endometrioid" "Endometrioid" "Missing"      "Missing"      "Endometrioid"
## [366] "Endometrioid" "Endometrioid" "Mixed"        "Missing"      "Endometrioid"
## [371] "Missing"      "Endometrioid" "Serous"       "Endometrioid" "Missing"     
## [376] "Endometrioid" "Endometrioid" "Endometrioid" "Mixed"        "Missing"     
## [381] "Missing"      "Mixed"        "Endometrioid" "Endometrioid" "Missing"     
## [386] "Endometrioid" "Endometrioid" "Endometrioid" "Endometrioid" "Endometrioid"
## [391] "Missing"      "Endometrioid" "Endometrioid" "Endometrioid" "Endometrioid"
## [396] "Endometrioid" "Missing"      "Endometrioid" "Endometrioid" "Missing"     
## [401] "Endometrioid" "Endometrioid" "Endometrioid" "Mixed"        "Missing"     
## [406] "Endometrioid" "Endometrioid" "Endometrioid" "Endometrioid" "Endometrioid"
## [411] "Missing"      "Missing"      "Missing"      "Serous"       "Endometrioid"
## [416] "Endometrioid" "Endometrioid" "Endometrioid" "Endometrioid" "Missing"     
## [421] "Missing"      "Endometrioid" "Missing"      "Mixed"        "Missing"     
## [426] "Endometrioid" "Endometrioid" "Endometrioid" "Missing"      "Endometrioid"
## [431] "Endometrioid" "Endometrioid" "Serous"       "Missing"      "Endometrioid"
## [436] "Endometrioid" "Missing"      "Serous"       "Serous"       "Mixed"       
## [441] "Endometrioid" "Missing"      "Endometrioid" "Serous"       "Endometrioid"
## [446] "Endometrioid" "Endometrioid" "Endometrioid" "Endometrioid" "Endometrioid"
## [451] "Missing"      "Endometrioid" "Endometrioid" "Endometrioid" "Serous"      
## [456] "Missing"      "Missing"      "Serous"       "Missing"      "Serous"      
## [461] "Missing"      "Missing"      "Missing"      "Endometrioid" "Missing"     
## [466] "Endometrioid" "Missing"      "Endometrioid" "Missing"      "Missing"     
## [471] "Endometrioid" "Missing"      "Missing"      "Endometrioid" "Endometrioid"
## [476] "Missing"      "Missing"      "Missing"      "Endometrioid" "Endometrioid"
## [481] "Missing"      "Missing"      "Missing"      "Mixed"        "Endometrioid"
## [486] "Missing"      "Endometrioid" "Endometrioid" "Missing"      "Serous"      
## [491] "Endometrioid" "Endometrioid" "Missing"      "Endometrioid" "Missing"     
## [496] "Endometrioid" "Missing"      "Endometrioid" "Endometrioid" "Endometrioid"
## [501] "Endometrioid" "Endometrioid" "Missing"      "Endometrioid" "Missing"     
## [506] "Endometrioid" "Missing"      "Missing"      "Endometrioid" "Serous"      
## [511] "Endometrioid" "Endometrioid" "Endometrioid" "Missing"      "Endometrioid"
## [516] "Endometrioid" "Endometrioid" "Missing"      "Endometrioid" "Endometrioid"
## [521] "Endometrioid" "Endometrioid" "Serous"       "Endometrioid" "Missing"     
## [526] "Serous"       "Missing"      "Endometrioid" "Missing"      "Missing"     
## [531] "Missing"      "Endometrioid" "Endometrioid" "Missing"      "Missing"     
## [536] "Endometrioid" "Missing"      "Endometrioid" "Endometrioid" "Missing"     
## [541] "Endometrioid" "Endometrioid"
```

```
# Endometrioid      Missing        Mixed       Serous 
#          304          172           13           53

ec_histology <- "Endometrioid"#"Serous" #

tcga_id_with_histo <- colData(e)[colData(e)$subtype_histology == ec_histology,] %>% rownames()

bound_nuHrRcpt_classif_mirna_filt2 <- bound_nuHrRcpt_classif %>% .[rownames(.) %>% substr(1,12) %in% substr(colnames(mir_t), 1,12),] %>% .[rownames(.) %>% substr(1,12) %in% substr(tcga_id_with_histo, 1,12),] 

mir_t3 <- rownames(bound_nuHrRcpt_classif_mirna_filt2) %>% 
  substr(1,12) %>%
  sapply(grep, colnames(mir_t)) %>%
  unlist %>%
  unique %>%
  mir_t[,.] %>%
  mutate_all(.funs = ~log(.+1, 2))

mir_n3 <- colnames(en) %>% 
  substr(1,12) %>%
  sapply(grep, colnames(mir_n)) %>%
  unlist %>%
  mir_n[,.] %>%
  mutate_all(.funs = ~log(.+1, 2))

e_sub <- substr(colnames(mir_t3), 1,12) %>% 
  sapply(grep,colnames(e)) %>%
  e[,.]
en_sub <- substr(colnames(mir_n3), 1,12) %>% 
  sapply(grep,colnames(en)) %>%
  en[,.]


#############
i=6
classif <- colnames(bound_nuHrRcpt_classif)[i]#"classif_ENSG00000082175"
vars <- c(classif, "seqPlate", "hosp")
colData_e_stdze <- colData(e_sub)[,vars]
dsgn <- model.matrix(~ colData(e_sub)[,classif] + colData(e_sub)[,"seqPlate"] + colData(e_sub)$hosp)
allDeGenes <- returnDeMirnaLimma(mir_t3, dsgn, colOfInt = 2, retAll=T)

logFCThr = 1
adjPvalThresh = 0.001

allDeGenes2 <- allDeGenes %>% rownames() %>% 
  as.numeric() %>% mir$miRNA_ID[.] %>%
  cbind(allDeGenes, mirna=.) %>%
  as_tibble() %>%
  mutate(mirna = as.character(mirna),
         mirna_label = ifelse(abs(logFC) > logFCThr & adj.P.Val < adjPvalThresh,
                              mirna,""))

rowIdxDeMirna <- rownames(allDeGenes)[1] %>% as.numeric
mir$miRNA_ID[rowIdxDeMirna]
```

```
## [1] "hsa-mir-146a"
```

```
#make a volcano plot
ggplot(allDeGenes2, 
       aes(x = logFC, y = -log(adj.P.Val, 10))) +
  theme_bw() + geom_vline(xintercept = c(-logFCThr,logFCThr), 
                          linetype="dashed", color = "grey") + 
  geom_hline(yintercept = -log(adjPvalThresh,10), 
             linetype="dashed", color = "grey") +
  geom_point(alpha=0.5)+ 
  geom_text_repel(aes(label=mirna_label))+
  theme_classic() +
  theme(text  = element_text(size=18), legend.title = element_blank()) + 
  labs(size = 14, x = expression(paste(log[2], "(miRNA in THRB_Hi/THRB_Low)")), 
       y = (expression(-log[10](q~value)))) + 
  annotate("segment", x=-1.3, y=2.6, xend=-1.2, yend=3.5, size=1) +
  
  annotate("rect", xmin=-Inf, xmax=-1, ymin=3, ymax=Inf, alpha=0.2, fill="red") +
  annotate("text", x=-1.5, y=2, label="miRNAs upregulated\nin endomet. tumors\nwith low THRB expr.", hjust = 0) +
    ggtitle("Diff miRNA expr in endometrial\ntumors with endometrioid histology")
```

## 7.3 Volcano plot showing miRNA differential expression in tumors with serous histology

```
ec_histology <- "Serous"

tcga_id_with_histo <- colData(e)[colData(e)$subtype_histology == ec_histology,] %>% rownames()

bound_nuHrRcpt_classif_mirna_filt2 <- bound_nuHrRcpt_classif %>% .[rownames(.) %>% substr(1,12) %in% substr(colnames(mir_t), 1,12),] %>% .[rownames(.) %>% substr(1,12) %in% substr(tcga_id_with_histo, 1,12),] 

mir_t3 <- rownames(bound_nuHrRcpt_classif_mirna_filt2) %>% 
  substr(1,12) %>%
  sapply(grep, colnames(mir_t)) %>%
  unlist %>%
  unique %>%
  mir_t[,.] %>%
  mutate_all(.funs = ~log(.+1, 2))

mir_n3 <- colnames(en) %>% 
  substr(1,12) %>%
  sapply(grep, colnames(mir_n)) %>%
  unlist %>%
  mir_n[,.] %>%
  mutate_all(.funs = ~log(.+1, 2))

e_sub <- substr(colnames(mir_t3), 1,12) %>% 
  sapply(grep,colnames(e)) %>%
  e[,.]
en_sub <- substr(colnames(mir_n3), 1,12) %>% 
  sapply(grep,colnames(en)) %>%
  en[,.]


#############
i=6
classif <- colnames(bound_nuHrRcpt_classif)[i]#"classif_ENSG00000082175"
vars <- c(classif, "seqPlate", "hosp")
colData_e_stdze <- colData(e_sub)[,vars]
dsgn <- model.matrix(~ colData(e_sub)[,classif] + colData(e_sub)[,"seqPlate"] + colData(e_sub)$hosp)
allDeGenes <- returnDeMirnaLimma(mir_t3, dsgn, colOfInt = 2, retAll=T)
```

```
## Coefficients not estimable: colData(e_sub)$hospEO
```

```
logFCThr = 1
adjPvalThresh = 0.001

allDeGenes2 <- allDeGenes %>% rownames() %>% 
  as.numeric() %>% mir$miRNA_ID[.] %>%
  cbind(allDeGenes, mirna=.) %>%
  as_tibble() %>%
  mutate(mirna = as.character(mirna),
         mirna_label = ifelse(abs(logFC) > logFCThr & adj.P.Val < adjPvalThresh,
                              mirna,""))

rowIdxDeMirna <- rownames(allDeGenes)[1] %>% as.numeric
mir$miRNA_ID[rowIdxDeMirna]
```

```
## [1] "hsa-mir-1237"
```

```
#make a volcano plot
ggplot(allDeGenes2, 
       aes(x = logFC, y = -log(adj.P.Val, 10))) +
  theme_bw() + geom_vline(xintercept = c(-logFCThr,logFCThr), 
                          linetype="dashed", color = "grey") + 
  geom_hline(yintercept = -log(adjPvalThresh,10), 
             linetype="dashed", color = "grey") +
  geom_point(alpha=0.5)+ 
  geom_text_repel(aes(label=mirna_label))+#aes(color=!inNeither & numHits > 3)) + 
  theme_classic() +
  theme(text  = element_text(size=18), legend.title = element_blank()) + 
  labs(size = 14, x = expression(paste(log[2], "(miRNA in THRB_Hi/THRB_Low)")), 
       y = (expression(-log[10](q~value)))) + 
  #annotate("segment", x=-0.7, y=0, xend=-1.15, yend=1.9, size=2, arrow=arrow(length = unit(0.03, "npc"), type="closed")) + 
  annotate("segment", x=-1.3, y=2.6, xend=-1.2, yend=3.5, size=1) +#, arrow=arrow(length = unit(0.03, "npc"), type="closed")) + 
  
  #annotate("text", x=-1.5, y=1, label="Upregulated in\nendomet. tumors\nwith low THRB expr.", hjust = 0) + 
  annotate("rect", xmin=-Inf, xmax=-1, ymin=3, ymax=Inf, alpha=0.2, fill="red") +
  annotate("text", x=-1.5, y=2, label="miRNAs upregulated\nin endomet. tumors\nwith low THRB expr.", hjust = 0) + 
  ggtitle("Diff miRNA expr in endometrial\ntumors with serous histology")
```

## 7.4 Expression of miRNA-146a across various tissue types

```
mir146a_expr_adj_nml <- as_tibble(list(id = 2, mir_expr = as.numeric(mir_n2[rowIdxDeMirna,])))

mir146a_expr <- as_tibble(list(id = bound_nuHrRcpt_classif_mirna_filt[,6], 
                          mir_expr = as.numeric(mir_t2[rowIdxDeMirna,]))) %>%
  bind_rows(mir146a_expr_adj_nml) %>%
  mutate(id = factor(id),
         id = recode(id, "0" = "Loss", #loss = THRB-low
         "1" = "Baseline", #THRB-high
         "2" = "Adj. Normal")) 

#statistical test to check difference between 2 groups
grp0 <- mir146a_expr %>% filter(as.character(id) == "Loss") %>% pull(mir_expr) %>% as.numeric()
grp1 <- mir146a_expr %>% filter(as.character(id) == "Baseline") %>% pull(mir_expr) %>% as.numeric()
grp2 <- mir146a_expr %>% filter(as.character(id) == "Adj. Normal") %>% pull(mir_expr) %>% as.numeric()

p1 <- wilcox.test(grp0, grp1) #p1$p.value 
p2 <- wilcox.test(grp1, grp2) 
p3 <- wilcox.test(grp0, grp2) 

p.adjust(c(p1$p.value, p2$p.value, p3$p.value), method = "BH")
```

```
## [1] 0.315592525 0.005456667 0.023650065
```

```
ggplot(mir146a_expr, aes(x=id, y=mir_expr, fill=id)) + 
  geom_violin() + 
  geom_jitter(pch=21, alpha=0.5) + 
  ylab("miRNA-146a expression") +
  xlab("") + 
  #ggtitle("Expression of miRNA-146a\nin endometrial cancer") + 
  scale_fill_manual(values=set2[c(1,2,4)]) +
  theme_classic() + 
  theme(legend.position="none", text=element_text(size=18, color="black"))
```

```
ggsave(filename = here::here("output/mir146.png"), width = 5.5, height = 4, units = "in")
```

## 7.5 Are the THRB-low tumors the same as the Type I endometrial cancers?

```
colDat_e_subst <- colData(e) %>% as_tibble %>% filter(subtype_histology == "Endometrioid" | subtype_histology == "Serous")#, Serous"))
#colData(e)$classif_ENSG00000151090
#varsToTest <- colData(e) %>% as_tibble %>% dplyr::select(patient, starts_with("subtype"))
ft <- fisher.test(colDat_e_subst$subtype_histology, colDat_e_subst$classif_ENSG00000151090)
table(colDat_e_subst$subtype_histology, colDat_e_subst$classif_ENSG00000151090)
```

```
##               
##                  0   1
##   Endometrioid  82 222
##   Serous         6  47
```

```
ft
```

```
## 
##  Fisher's Exact Test for Count Data
## 
## data:  colDat_e_subst$subtype_histology and colDat_e_subst$classif_ENSG00000151090
## p-value = 0.01501
## alternative hypothesis: true odds ratio is not equal to 1
## 95 percent confidence interval:
##  1.170583 8.574699
## sample estimates:
## odds ratio 
##   2.886447
```

The THRB-low tumors (with better survival) are more likely to be endometrioid (type I), pvalue = 0.015, OR = 2.886.

# 8 THRB expression in other cancers

we look at THRB expression patterns in other cancer types (in both tumor and normal tissue) and ask whether THRB expression is predictive of survival in other cancers.

```
brca <- readRDS(here("data", "TCGA-BRCA-exp.rds")) %>% clean_dataset()
```

```
## [1] 56716  1222
## [1] "Dimensions after FFPE removed:"
## [1] 56716  1206
## [1] "Dimensions of tumor and adj nml dataframes:"
## [1] 56716  1086
## [1] 56716   113
## [1] "Dimensions of tumor and adj nml dataframes (duplicates removed):"
## [1] 56716  1081
## [1] 56716   113
## [1] "Dimensions of tumor and adj nml dataframes (duplicates removed + pts with NA values removed):"
## [1] 56716  1081
## [1] 56716   113
## [1] "Dimensions of tumor and adj nml dataframes (duplicates removed + pts with NA values removed + 20% filter):"
## [1] 37798  1081
## [1] 38482   113
## [1] "Dimensions of tumor and adj nml dataframes (duplicates removed + pts with NA values removed + 20% filter + intersection):"
## [1] 36715  1081
## [1] 36715   113
```

```
luad <- readRDS(here("data", "TCGA-LUAD-exp.rds")) %>% clean_dataset()
```

```
## [1] 56716   594
## [1] "Dimensions after FFPE removed:"
## [1] 56716   583
## [1] "Dimensions of tumor and adj nml dataframes:"
## [1] 56716   522
## [1] 56716    59
## [1] "Dimensions of tumor and adj nml dataframes (duplicates removed):"
## [1] 56716   513
## [1] 56716    59
## [1] "Dimensions of tumor and adj nml dataframes (duplicates removed + pts with NA values removed):"
## [1] 56716   513
## [1] 56716    59
## [1] "Dimensions of tumor and adj nml dataframes (duplicates removed + pts with NA values removed + 20% filter):"
## [1] 38226   513
## [1] 36251    59
## [1] "Dimensions of tumor and adj nml dataframes (duplicates removed + pts with NA values removed + 20% filter + intersection):"
## [1] 35380   513
## [1] 35380    59
```

```
lusc <- readRDS(here("data", "TCGA-LUSC-exp.rds")) %>% clean_dataset()
```

```
## [1] 56716   551
## [1] "Dimensions after FFPE removed:"
## [1] 56716   551
## [1] "Dimensions of tumor and adj nml dataframes:"
## [1] 56716   502
## [1] 56716    49
## [1] "Dimensions of tumor and adj nml dataframes (duplicates removed):"
## [1] 56716   501
## [1] 56716    49
## [1] "Dimensions of tumor and adj nml dataframes (duplicates removed + pts with NA values removed):"
## [1] 56716   501
## [1] 56716    49
## [1] "Dimensions of tumor and adj nml dataframes (duplicates removed + pts with NA values removed + 20% filter):"
## [1] 39216   501
## [1] 38451    49
## [1] "Dimensions of tumor and adj nml dataframes (duplicates removed + pts with NA values removed + 20% filter + intersection):"
## [1] 36698   501
## [1] 36698    49
```

```
brca_n <- brca$en
brca <- brca$et

luad_n <- luad$en
luad <- luad$et

lusc_n <- lusc$en
lusc <- lusc$et

ucec_n <- en
ucec <- e

names_of_dfs <- c("ucec_n","brca_n", "luad_n", "lusc_n", "brca", "luad", "lusc", "ucec")

esr1 = "ENSG00000091831"
ar = "ENSG00000169083"
pr = "ENSG00000082175"
thrb = "ENSG00000151090"
thra = "ENSG00000126351"
recepts = c(esr1, ar, pr, thrb, thra)
```

## 8.1 What does THRB receptor expression look like in other cancers?

```
thrb_df <- lapply(names_of_dfs, function(x) assay_to_df(eval(parse(text = x)), tum_type = x, gene = thrb)) %>%
  do.call(rbind, .) %>% 
  mutate(class_temp = substr(tum_type, start = nchar(tum_type)-1, stop=nchar(tum_type)),
                   class = ifelse(class_temp == "_n", "adj_nml", "tumor")) %>%
  select(-class_temp) %>%
  mutate(tum_type2 = substr(tum_type, 1,4),
         class=factor(class),
         tum_name = factor(tum_type2, levels = c("ucec", "brca", "luad", "lusc"),
                           labels = c("Uterus", "Breast", "Lung Adeno", "Lung Squam")))


ggplot(thrb_df, aes(x=tum_name, y=ENSG00000151090, color=class)) +
  scale_color_manual(values=set2[4:3]) + 
  geom_boxplot(fill=NA, outlier.shape = NA, size=1, alpha=0.8) + 
  theme_classic() + 
  geom_point(pch = 21, alpha=0.8,
             position = position_jitterdodge(jitter.height = 0, jitter.width = 0.5)) + 
  xlab("") + ylab("THRB expression (log2(TPM))")
```

## 8.2 Run receptLoss on other cancer types and look at THRB expression here

```
brca_res <- receptLoss(exprMatrNml=assay(brca_n), exprMatrTum=assay(brca), nSdBelow=2, minPropPerGroup=0.2)
luad_res <- receptLoss(exprMatrNml=assay(luad_n), exprMatrTum=assay(luad), nSdBelow=2, minPropPerGroup=0.2)
lusc_res <- receptLoss(exprMatrNml=assay(lusc_n), exprMatrTum=assay(lusc), nSdBelow=2, minPropPerGroup=0.2)

receptLoss::plotReceptLoss(exprMatrNml = assay(brca_n), exprMatrTum = assay(brca), rldf = brca_res, geneName = thrb, clrs = set2[4:3])
```

```
receptLoss::plotReceptLoss(exprMatrNml = assay(luad_n), exprMatrTum = assay(luad), rldf = luad_res, geneName = thrb, clrs = set2[4:3])
```

```
receptLoss::plotReceptLoss(exprMatrNml = assay(lusc_n), exprMatrTum = assay(lusc), rldf = lusc_res, geneName = thrb, clrs = set2[4:3])
```

## 8.3 Is there a difference in survival between high and low groups based on THRB expression in other tumors? Classify the tumors first, then perform K-M survival analysis

```
colData(brca) <- bindClassToColData(cdat = colData(brca), returnClassif(brca, brca_res, thrb))  %>%
  bindClassToColData(obj = calc_days_last_fu(colData(brca)$days_to_death,
                                             colData(brca)$days_to_last_follow_up)) %>% 
  bindClassToColData(calc_vital_stat(vital_stat = colData(brca)$vital_status, .$days_last_fu))

colData(luad) <- bindClassToColData(cdat = colData(luad), 
                                    obj = returnClassif(luad, luad_res, thrb)) %>%
  bindClassToColData(obj = calc_days_last_fu(colData(luad)$days_to_death,
                                             colData(luad)$days_to_last_follow_up)) %>% 
  bindClassToColData(calc_vital_stat(vital_stat = colData(luad)$vital_status, .$days_last_fu))

colData(lusc) <- bindClassToColData(cdat = colData(lusc), 
                                    obj = returnClassif(lusc, lusc_res, thrb)) %>%
  bindClassToColData(obj = calc_days_last_fu(colData(lusc)$days_to_death,
                                             colData(lusc)$days_to_last_follow_up)) %>% 
  bindClassToColData(calc_vital_stat(vital_stat = colData(lusc)$vital_status, .$days_last_fu))


plotSurvAndReturnStat(timeToEvent = colData(brca)$days_last_fu/365.25, alive0dead1 = colData(brca)$vital_stat, classif = colData(brca)$classif_ENSG00000151090, surv.col = set2[1:2], gnNm = "BRCA survival based on THRB expression")
```

```
## Call:
## survdiff(formula = Surv(timeToEvent, alive0dead1) ~ classif)
## 
## n=1079, 2 observations deleted due to missingness.
## 
##             N Observed Expected (O-E)^2/E (O-E)^2/V
## classif=0 809       71     72.9     0.051     0.189
## classif=1 270       29     27.1     0.138     0.189
## 
##  Chisq= 0.2  on 1 degrees of freedom, p= 0.7
```

```
## Call:
## survdiff(formula = Surv(timeToEvent, alive0dead1) ~ classif)
## 
## n=1079, 2 observations deleted due to missingness.
## 
##             N Observed Expected (O-E)^2/E (O-E)^2/V
## classif=0 809       71     72.9     0.051     0.189
## classif=1 270       29     27.1     0.138     0.189
## 
##  Chisq= 0.2  on 1 degrees of freedom, p= 0.7
```

```
plotSurvAndReturnStat(timeToEvent = colData(luad)$days_last_fu/365.25, alive0dead1 = colData(luad)$vital_stat, classif = colData(luad)$classif_ENSG00000151090, surv.col = set2[1:2], gnNm = "LUAD survival based on THRB expression")
```

```
## Call:
## survdiff(formula = Surv(timeToEvent, alive0dead1) ~ classif)
## 
## n=504, 9 observations deleted due to missingness.
## 
##             N Observed Expected (O-E)^2/E (O-E)^2/V
## classif=0 308       97    108.1      1.13      3.06
## classif=1 196       75     63.9      1.92      3.06
## 
##  Chisq= 3.1  on 1 degrees of freedom, p= 0.08
```

```
## Call:
## survdiff(formula = Surv(timeToEvent, alive0dead1) ~ classif)
## 
## n=504, 9 observations deleted due to missingness.
## 
##             N Observed Expected (O-E)^2/E (O-E)^2/V
## classif=0 308       97    108.1      1.13      3.06
## classif=1 196       75     63.9      1.92      3.06
## 
##  Chisq= 3.1  on 1 degrees of freedom, p= 0.08
```

```
plotSurvAndReturnStat(timeToEvent = colData(lusc)$days_last_fu/365.25, alive0dead1 = colData(lusc)$vital_stat, classif = colData(lusc)$classif_ENSG00000151090, surv.col = set2[1:2], gnNm = "LUSC survival based on THRB expression")
```

```
## Call:
## survdiff(formula = Surv(timeToEvent, alive0dead1) ~ classif)
## 
## n=495, 6 observations deleted due to missingness.
## 
##             N Observed Expected (O-E)^2/E (O-E)^2/V
## classif=0 249       88     90.3    0.0575     0.115
## classif=1 246       93     90.7    0.0572     0.115
## 
##  Chisq= 0.1  on 1 degrees of freedom, p= 0.7
```

```
## Call:
## survdiff(formula = Surv(timeToEvent, alive0dead1) ~ classif)
## 
## n=495, 6 observations deleted due to missingness.
## 
##             N Observed Expected (O-E)^2/E (O-E)^2/V
## classif=0 249       88     90.3    0.0575     0.115
## classif=1 246       93     90.7    0.0572     0.115
## 
##  Chisq= 0.1  on 1 degrees of freedom, p= 0.7
```

# 9 Session Information

```
sessionInfo()
```

```
## R version 3.6.0 (2019-04-26)
## Platform: x86_64-apple-darwin15.6.0 (64-bit)
## Running under: macOS Mojave 10.14.6
## 
## Matrix products: default
## BLAS:   /Library/Frameworks/R.framework/Versions/3.6/Resources/lib/libRblas.0.dylib
## LAPACK: /Library/Frameworks/R.framework/Versions/3.6/Resources/lib/libRlapack.dylib
## 
## locale:
## [1] en_US.UTF-8/en_US.UTF-8/en_US.UTF-8/C/en_US.UTF-8/en_US.UTF-8
## 
## attached base packages:
## [1] parallel  stats4    stats     graphics  grDevices utils     datasets 
## [8] methods   base     
## 
## other attached packages:
##  [1] receptLoss_0.99.9           patchwork_1.0.0            
##  [3] ggrepel_0.8.2               fgsea_1.10.1               
##  [5] Rcpp_1.0.4.6                limma_3.40.6               
##  [7] ggdendro_0.1-20             survival_3.1-12            
##  [9] janitor_2.0.0               forcats_0.5.0              
## [11] stringr_1.4.0               dplyr_0.8.5                
## [13] purrr_0.3.3                 readr_1.3.1                
## [15] tidyr_1.0.2                 tibble_3.0.0               
## [17] ggplot2_3.3.0               tidyverse_1.3.0            
## [19] biomaRt_2.40.5              mclust_5.4.5               
## [21] here_0.1                    RColorBrewer_1.1-2         
## [23] SummarizedExperiment_1.14.1 DelayedArray_0.10.0        
## [25] BiocParallel_1.18.1         matrixStats_0.56.0         
## [27] Biobase_2.44.0              GenomicRanges_1.36.1       
## [29] GenomeInfoDb_1.20.0         IRanges_2.18.3             
## [31] S4Vectors_0.24.4            BiocGenerics_0.32.0        
## 
## loaded via a namespace (and not attached):
##  [1] nlme_3.1-145           bitops_1.0-6           fs_1.4.1              
##  [4] lubridate_1.7.8        bit64_0.9-7            progress_1.2.2        
##  [7] httr_1.4.1             rprojroot_1.3-2        tools_3.6.0           
## [10] backports_1.1.6        utf8_1.1.4             R6_2.4.1              
## [13] DBI_1.1.0              colorspace_1.4-1       withr_2.1.2           
## [16] GGally_1.5.0           gridExtra_2.3          tidyselect_1.0.0      
## [19] prettyunits_1.1.1      bit_1.1-15.2           compiler_3.6.0        
## [22] cli_2.0.2              rvest_0.3.5            xml2_1.3.1            
## [25] labeling_0.3           bookdown_0.18          scales_1.1.0          
## [28] digest_0.6.25          rmarkdown_2.1          XVector_0.24.0        
## [31] pkgconfig_2.0.3        htmltools_0.4.0        dbplyr_1.4.2          
## [34] rlang_0.4.5            readxl_1.3.1           rstudioapi_0.11       
## [37] RSQLite_2.2.0          farver_2.0.3           generics_0.0.2        
## [40] jsonlite_1.6.1         RCurl_1.98-1.1         magrittr_1.5          
## [43] GenomeInfoDbData_1.2.1 Matrix_1.2-18          munsell_0.5.0         
## [46] fansi_0.4.1            lifecycle_0.2.0        stringi_1.4.6         
## [49] yaml_2.2.1             snakecase_0.11.0       MASS_7.3-51.5         
## [52] zlibbioc_1.30.0        plyr_1.8.6             grid_3.6.0            
## [55] blob_1.2.1             crayon_1.3.4           lattice_0.20-41       
## [58] splines_3.6.0          haven_2.2.0            hms_0.5.3             
## [61] knitr_1.28             pillar_1.4.3           reshape2_1.4.4        
## [64] fastmatch_1.1-0        reprex_0.3.0           XML_3.99-0.3          
## [67] glue_1.4.0             evaluate_0.14          data.table_1.12.8     
## [70] modelr_0.1.6           vctrs_0.2.4            rmdformats_0.3.7      
## [73] cellranger_1.1.0       gtable_0.3.0           reshape_0.8.8         
## [76] assertthat_0.2.1       xfun_0.12              broom_0.5.5           
## [79] AnnotationDbi_1.46.1   memoise_1.1.0          ellipsis_0.3.0
```

## 9.1 Supplementary scripts

The following scripts are found in the file `2019_endomet_smry_suppl.R`.

```
orderSeqPlate <- function(x){
  x %>% 
    group_by(seqPlate) %>%
    dplyr::count(seqPlate) %>% 
    arrange(-n)
}

countSeqPlate <- function(x, sqPlates){
  x %>%
    group_by(bin_expr, seqPlate, ensembl_gene_id) %>%
    dplyr::count(seqPlate) %>%
    ungroup() %>%
    mutate(seqPlate = factor(seqPlate, levels=sqPlates)) %>%
    mutate(bin_expr = factor(bin_expr, c(1,0)))
}

#scripts for doing GSEA with Fisher's exact test
#R package: gSEAfish - Set enrichment analysis using Fisher's exact test
#also provides visualization tools
 
genesetEnrichmentFisher <- function(B, Targ, GS.GS, GS.names){ 
  #calculates the fisher exact t test for enrichment in a particular set of
  #target gene entrez IDs (T) among the number of background genes (B). GS.GS
  #takes the form "hGMT$genesets", where hGMT is a variable that stores the .gmt
  #file object downloaded from the msigdb website that was read in using the
  #GMT() function. GS.names takes the form "hGMT$geneset.names"
  
  #1. Initialize an empty dataframe
  t <- unlist(fisher.test(matrix(data = c(1,4,6,2), nrow = 2), conf.int = T))
  colNam <- c(names(t), "geneset.names", "MatchesBtwTargAndPathway", "NumbGenesInMsigdbPathway", "TargLen", "BackgroundLen")
  fisherDf <- data.frame(matrix(NA, nrow=1, ncol=13))
  names(fisherDf) <- colNam
  
  TargLen <- length(Targ)
  #2. Loop through each geneset and calculate pvalues
  for(i in 1:length(GS.GS)){
    pathwayEntrezIDs <- GS.GS[[i]] #these are characters
    M <- length(intersect(Targ, pathwayEntrezIDs)) #number of overlapping genes
    P <- length(pathwayEntrezIDs) #numb of genes in pathway
    conting.matrix <- matrix(c(M, TargLen-M, P-M, B-TargLen-P+M), nrow = 2, byrow = TRUE) #create contigency matrix
    fisherTest <- fisher.test(conting.matrix, conf.int = T)
    fisherDf <- rbind(fisherDf, c(unlist(fisherTest), GS.names[[i]], M, P, TargLen, B))
  }
  fisherDf = fisherDf[-1,]   #remove the row of NA's (in row 1)
  fisherDf$bhPval <- p.adjust(fisherDf$p.value, method = "BH")
  fisherDf$bonferroni <- p.adjust(fisherDf$p.value, method = "bonferroni")
  fisherDf$neg.log10.bhPval <- -log10(as.numeric(fisherDf$bhPval))
  fisherDf$dataset <-
    unlist(lapply(strsplit(fisherDf$geneset.names, "_"), function(x) x[1]))
  fisherDf <- fisherDf[order(fisherDf$bhPval),]
  #rank by bhPval pbal
  return(fisherDf)
}


forestplot <- function(d, title, xlab=expression(log[10] *"(Odds Ratio)"), ylab="Pathway", size=12){
  p <- ggplot(d, aes(x=x, y=y, ymin=ylo, ymax=yhi)) +
    geom_pointrange(size = 1.25) +
    coord_flip() +
    #geom_hline(aes(x=0, yintercept = 1)) +
    ylab(xlab) +
    xlab(ylab) + #switch because of the coord_flip() above
    ggtitle(title) +
    scale_y_log10() +
    theme_classic() +
    geom_text(aes(label=numbMatches)) +
    guides(alpha=FALSE) + 
    theme(axis.text.x=element_text(size=size), axis.text.y = element_text(size = 7), legend.text = element_text(size = 12))
  return(p)
}

createForestDF <- function(data, title){
  d <- data.frame(x=data$geneset.names, y=as.numeric(data$`estimate.odds ratio`), ylo = as.numeric(data$conf.int1), yhi = as.numeric(data$conf.int2), pval=as.numeric(data$p.value), numbGeneInPway = data$NumbGenesInMsigdbPathway,
                  numbMatches=data$MatchesBtwTargAndPathway)
  d$x <-factor(d$x, levels=d[order(-d$y), "x"])
  return(forestplot(d, title, xlab=expression("Odds Ratio("*log[10] *" scale)")))
}

gseWrapper <- function(B, Targ, genesets, geneset.names, qval, OR){
  #dir can equal "up" or "down" for up or down regulated, respectivtly
  res_gse <- genesetEnrichmentFisher(B = B, Targ = Targ, GS.GS = genesets, GS.names=geneset.names)
  res_gse_filt <- res_gse[as.numeric(res_gse$bhPval) < qval & as.numeric(res_gse$`estimate.odds ratio`) > OR & as.numeric(res_gse$conf.int1) > OR,]
  return(res_gse_filt)
}


readHmKgRctmepathways <- function(gsea_path){
  hallmark <- GSA.read.gmt(paste0(gsea_path,"h.all.v5.1.entrez.gmt")) #50 genesets
  kegg <- GSA.read.gmt(paste0(gsea_path,"c2.cp.kegg.v5.1.entrez.gmt")) #185 genesets
  reactome <- GSA.read.gmt(paste0(gsea_path,"c2.cp.reactome.v5.1.entrez.gmt")) #673 genesets
  hallmarkKeggReactome <- list(genesets = c(hallmark$genesets, kegg$genesets, reactome$genesets), geneset.names =
                                 c(hallmark$geneset.names, kegg$geneset.names, reactome$geneset.names), geneset.descriptions =
                                 c(hallmark$geneset.descriptions, kegg$geneset.descriptions, reactome$geneset.descriptions))
  return(hallmarkKeggReactome)
}

ensgToEntrez2 <- function(ensg, geneMap){
  g <- geneMap %>% 
    dplyr::select(-ucsc) %>% 
    distinct %>% 
    filter(ensembl_gene_id %in% ensg)
  return(g)
}


geneSymbToEnsg <- function(geneSymb){
  ensembl = useMart("ensembl",dataset="hsapiens_gene_ensembl")
  geneEntrez <- getBM(attributes = c("ensembl_gene_id", "hgnc_symbol", "entrezgene"),
                      filters = "hgnc_symbol",
                      values = geneSymb,
                      mart = ensembl)
  return(geneEntrez)
}


twoSdBelowMean <- function(mn, sd){
  twosdblw <- mn - 2*sd
  return(twosdblw)
}

twoSdAboveMean <- function(mn, sd){
  twosdabv <- mn + 2*sd
  return(twosdabv)
}

threeSdBelowMean <- function(mn, sd){
  threesdblw <- mn - 3*sd
  return(threesdblw)
}

threeSdAboveMean <- function(mn, sd){
  threesdabv <- mn + 3*sd
  return(threesdabv)
}
fourSdBelowMean <- function(mn, sd){
  fsdblw <- mn - 4*sd
  return(fsdblw)
}

fourSdAboveMean <- function(mn, sd){
  fsdabv <- mn + 4*sd
  return(fsdabv)
}

poundToKg <- function(lb){
  kg_per_lb = 2.2046226218
  return(lb/kg_per_lb)
}

inToCm <- function(inches){
  cm_per_in = 2.54
  return(cm_per_in*inches)
}


plotSurvAndReturnStat <- function(timeToEvent, alive0dead1, classif, surv.col, gnNm=""){
  fit <- survfit(Surv(timeToEvent, alive0dead1) ~ classif)
  fit.stat <- survdiff(Surv(timeToEvent, alive0dead1) ~ classif)
  print(fit.stat)
  #650x250
  p <- GGally::ggsurv(fit, back.white = T, surv.col = surv.col) + 
    ylim(c(0,1)) + theme_classic() + 
    ggtitle(gnNm) +
    theme(aspect.ratio = 1, legend.title=element_blank(), 
          text = element_text(size=25, color = "black"), 
          axis.title=element_blank(),
          plot.title =element_text(face="italic")) +
  #add the number of patients
  annotate("text", x = 1, y = .35, label = paste0(names(fit.stat$n)[1], ", N = ",fit.stat$n[1])) +
  annotate("text", x = 1, y = .15, label = paste0(names(fit.stat$n)[2], ", N = ",fit.stat$n[2]))

  print(p)
  return(fit.stat)
}


returnDeGenesLimma <- function(expr, design, colOfInt, pval=1, logFC=0, retAll=F){
  fit_t <- lmFit(object = assay(expr), design = design)
  ebfit_t <- eBayes(fit_t)
  if(retAll == T){
    topTableRes_t <- topTable(ebfit_t, coef = colOfInt, number = Inf) 
    return(topTableRes_t)
  }
  
  topTableRes_t <- topTable(ebfit_t, coef = colOfInt, number = nrow(assay(expr)), adjust.method = "BH", p.value = pval, lfc = logFC) 
  return(topTableRes_t)
}

bindClassToColData <- function(cdat, obj){
  #if the columns exist, replace them
  #if the column does not exist, add it
  cno <- colnames(obj)
  for(col in 1:ncol(obj)){
    cdat$temp1 <- obj[,col] #add a temporary column from obj
    if(cno[col] %in% colnames(cdat)){
      #the column does exist, so replace it
      cdat[, cno[col]] <- NULL
    }
    colnames(cdat)[which(colnames(cdat) == "temp1")] <- cno[col]
  }
  return(cdat)
}


plotGeneHist2 <- function(exprNml, exprTum, isof, title, clrs){

  tidyDf <- as.data.frame(cbind(as.numeric(c(exprTum[isof,], exprNml[isof,])),
                                as.factor(c(rep("tumor",ncol(exprTum)), rep("normal",ncol(exprNml))))),
                          stringsAsFactors=FALSE)
  colnames(tidyDf) <- c("expr", "type")
  print(mean(exprNml[isof,]))
  print(twoSdBelowMean(mn=mean(exprNml[isof,]),sd = sd(exprNml[isof,])))
  expr <- type <- ..density.. <- NULL # Setting the variables to NULL first
  p1 <- ggplot(tidyDf, aes(x=expr, color=as.factor(type),
                           fill=as.factor(type),
                           group=as.factor(type))) +
    theme_classic() +
    geom_histogram(data=subset(tidyDf,type == 2),
                   alpha=0.2, aes(y=..density..), binwidth = .2, 
                   color=clrs[2], fill=clrs[2]) +
    geom_rug(alpha=0.3, show.legend=FALSE) +
    scale_fill_manual(values=clrs) + 
    scale_color_manual(values=clrs) + 
    theme(axis.title=element_blank(),
          axis.text.y=element_blank(),
          axis.ticks.y=element_blank(),
          axis.line.y=element_blank(),
          legend.position="none",
          text = element_text(size=35, color="black"),
          plot.title=element_text(face="italic")) + coord_fixed(ratio=15) + 
    xlim(c(-0.1, 10)) +
    ylim(c(0,.63))+
    ggtitle(title) + 
    stat_function(fun="dnorm", colour=clrs[1],
                  args=list(mean=mean(exprNml[isof,]),
                            sd=sd(exprNml[isof,])), linetype="dashed", size=1.5) +
    geom_vline(size=2, alpha=0.8, color=clrs[1], xintercept = twoSdBelowMean(mn=mean(exprNml[isof,]),sd = sd(exprNml[isof,])))
    print(p1)
}


plotGene_bothHist <- function(exprNml, exprTum, isof, title, clrs){

  tidyDf <- as.data.frame(cbind(as.numeric(c(exprTum[isof,], exprNml[isof,])),
                                as.factor(c(rep("tumor",ncol(exprTum)), rep("normal",ncol(exprNml))))),
                          stringsAsFactors=FALSE)
  colnames(tidyDf) <- c("expr", "type")
  print(mean(exprNml[isof,]))
  print(twoSdBelowMean(mn=mean(exprNml[isof,]),sd = sd(exprNml[isof,])))
  expr <- type <- ..density.. <- NULL # Setting the variables to NULL first
  p1 <- ggplot(tidyDf, aes(x=expr, color=as.factor(type),
                           fill=as.factor(type),
                           group=as.factor(type))) +
    theme_classic() +
    geom_histogram(data=subset(tidyDf,type == 1),
                   alpha=0.2, aes(y=..density..), binwidth = .2, 
                   color=clrs[1], fill=clrs[1]) +
    geom_histogram(data=subset(tidyDf,type == 2),
                   alpha=0.2, aes(y=..density..), binwidth = .2, 
                   color=clrs[2], fill=clrs[2]) +
    geom_rug(alpha=0.3, show.legend=FALSE) +
    scale_fill_manual(values=clrs) + 
    scale_color_manual(values=clrs) + 
    theme(axis.title=element_blank(),
          axis.text.y=element_blank(),
          axis.ticks.y=element_blank(),
          axis.line.y=element_blank(),
          legend.position="none",
          text = element_text(size=35, color="black"),
          plot.title=element_text(face="italic")) + coord_fixed(ratio=15) + 
    xlim(c(-0.1, 10)) +
    ggtitle(title) + 
    geom_vline(size=2, alpha=0.8, color=clrs[1], xintercept = twoSdBelowMean(mn=mean(exprNml[isof,]),sd = sd(exprNml[isof,])))
  print(p1)
}


plotGene_oneHist <- function(exprNml, exprTum, isof, title, clrs, vert_line = TRUE){
  
  tidyDf <- as.data.frame(cbind(as.numeric(c(exprTum[isof,], exprNml[isof,])),
                                as.factor(c(rep("tumor",ncol(exprTum)), rep("normal",ncol(exprNml))))),
                          stringsAsFactors=FALSE)
  colnames(tidyDf) <- c("expr", "type")
  print(mean(exprNml[isof,]))
  print(twoSdBelowMean(mn=mean(exprNml[isof,]),sd = sd(exprNml[isof,])))
  expr <- type <- ..density.. <- NULL # Setting the variables to NULL first
  p1 <- ggplot(tidyDf, aes(x=expr, color=as.factor(type),
                           fill=as.factor(type),
                           group=as.factor(type))) +
    theme_classic() +
    geom_histogram(data=subset(tidyDf,type == 1),
                   alpha=0.2, aes(y=..density..), binwidth = .2, 
                   color=clrs[1], fill=clrs[1]) +
     scale_fill_manual(values=clrs) + 
    scale_color_manual(values=clrs) + 
    theme(axis.title=element_blank(),
          axis.text.y=element_blank(),
          axis.ticks.y=element_blank(),
          axis.line.y=element_blank(),
          legend.position="none",
          text = element_text(size=35, color="black"),
          plot.title=element_text(face="italic")) + coord_fixed(ratio=15) + 
    xlim(c(-0.1, 10)) +
    ggtitle(title) #+ 
  if(vert_line == TRUE){
    p1 <- p1 + geom_vline(size=2, alpha=0.8, color=clrs[1], xintercept = twoSdBelowMean(mn=mean(exprNml[isof,]),sd = sd(exprNml[isof,])))
  } 
  print(p1)
}


plotGene_oneHistNmlCurve <- function(exprNml, exprTum, isof, title, clrs, vert_line = TRUE, histogram = TRUE){
  
  tidyDf <- as.data.frame(cbind(as.numeric(c(exprTum[isof,], exprNml[isof,])),
                                as.factor(c(rep("tumor",ncol(exprTum)), rep("normal",ncol(exprNml))))),
                          stringsAsFactors=FALSE)
  colnames(tidyDf) <- c("expr", "type")
  print(mean(exprNml[isof,]))
  print(twoSdBelowMean(mn=mean(exprNml[isof,]),sd = sd(exprNml[isof,])))
  expr <- type <- ..density.. <- NULL # Setting the variables to NULL first
  p1 <- ggplot(tidyDf, aes(x=expr, color=as.factor(type),
                           fill=as.factor(type),
                           group=as.factor(type))) +
    theme_classic() +
    scale_fill_manual(values=clrs) + 
    scale_color_manual(values=clrs) + 
    theme(axis.title=element_blank(),
          axis.text.y=element_blank(),
          axis.ticks.y=element_blank(),
          axis.line.y=element_blank(),
          legend.position="none",
          text = element_text(size=35, color="black"),
          plot.title=element_text(face="italic")) + coord_fixed(ratio=15) + 
    xlim(c(-0.1, 10)) +
    ggtitle(title) + 
    stat_function(fun="dnorm", colour=clrs[1],
                  args=list(mean=mean(exprNml[isof,]),
                            sd=sd(exprNml[isof,])), linetype="dashed", size=1.5)
    
  if(histogram == TRUE){
    p1 <- p1 + geom_histogram(data=subset(tidyDf,type == 1),
                              alpha=0.2, aes(y=..density..), binwidth = .2, 
                              color=clrs[1], fill=clrs[1])
  }
  if(vert_line == TRUE){
    p1 <- p1 + geom_vline(size=2, alpha=0.8, color=clrs[1], xintercept = twoSdBelowMean(mn=mean(exprNml[isof,]),sd = sd(exprNml[isof,])))
  }

  print(p1)
}


pvalFxn <- function(survDifObj){
  p.val <- 1 - pchisq(survDifObj$chisq, length(survDifObj$n) - 1)
  return(p.val)
}


sig2star = function(s, breaks=c(-Inf, 0.0001,0.001,0.01,1), labels=c("***","**","*","")) {
  r <- s
  r[] <- as.character(cut(s, breaks=breaks, labels)) 
  r[is.na(r)] <- ""
  return(r)
}


#' Cluster and Reorder Rows and Columns
#'
#' Clusters the rows and columns of a numeric matrix using hclust
#'
#' @param matr matrix to be clustered
#' @param dimen Indicates the dimension that the clustering is performed in. One of either "row", "column", or "both". 
#'
#' @return returns a matrix of clustered values by either row, column, or both, 

clusterAndReorder <- function(matr, dimen="both"){
  #dimen can be one of row, column or both
  if(dimen == "row"){
    hcl <- hclust(dist(matr))
    matr.new <- matr[hcl$order,]
    return(matr.new)
  } else if(dimen == "column"){
    hcl <- hclust(dist(t(matr)))
    matr.new <- matr[,hcl$order]
    return(matr.new)
  } else if(dimen == "both"){
    hcl.r <- hclust(dist(matr))
    hcl.c <- hclust(dist(t(matr)))
    matr.new <- matr[hcl.r$order, hcl.c$order]
    return(matr.new)
  }
}


set2 <- RColorBrewer::brewer.pal(n = 8, "Set2")
bw <- RColorBrewer::brewer.pal(n = 9, "Greys")

ggBar <- list(
  theme_classic(),
  theme(legend.position="top",
        axis.ticks = element_blank(),
        axis.text = element_text(size=18, color="black"),
        axis.text.x = element_text(angle = 90, hjust=1,vjust=0.5),
        axis.title = element_text(size=22)),
  scale_y_continuous(expand = c(0,0)))

ggHist <- list(
  theme_classic(),
  theme(legend.position="top",
        axis.text = element_text(size=24)),
  scale_y_continuous(expand = c(0,0)))


ggSeqPlate = list(
  geom_bar(stat="identity"),
  facet_grid(.~ensembl_gene_id),
  theme_classic(), 
  theme(axis.text=element_text(size=10),
        axis.text.x = element_text(angle=90, hjust = 1, vjust=0.5),
        axis.text.y = element_text(size=16)),
  scale_fill_manual(values=set2[2:1]))


clean_dataset <- function(e){
  
  #returns adj normal and tumor dataframes
  print(dim(e))
  
  #remove ffpe samples
  e <- e[,!e$is_ffpe]
  print("Dimensions after FFPE removed:")
  print(dim(e)) 
  
  #add technical variables
  colData(e)$hosp <- substr(rownames(colData(e)), 6,7)
  colData(e)$seqPlate <- substr(rownames(colData(e)), 22,25)
  
  ###remove all metaData columns that are NA
  colData(e) <- colData(e)[,colSums(is.na(colData(e)))<nrow(colData(e))]
  
  ##TPM normalization, then log2(tpm+1) transform
  eu <- assay(e)
  eu <- scale(eu, center=FALSE, scale=colSums(eu)) * 1000000
  eu <- log(eu+1, 2)
  assay(e) <- eu
  
  #separate tumor and adjacent normal samples
  et <- e[,substr(colnames(e),14,15) == "01"] 
  en <- e[,substr(colnames(e),14,15) == "11"]
  print("Dimensions of tumor and adj nml dataframes:")
  print(dim(et)); print(dim(en))
  
  #remove duplicates
  et <- et[,!duplicated(substr(colnames(et),1,12))] 
  en <- en[,!duplicated(substr(colnames(en),1,12))] 
  print("Dimensions of tumor and adj nml dataframes (duplicates removed):")
  print(dim(et)); print(dim(en))

  #remove patients with NA values
  et <- et[,!apply(assay(et), 2, anyNA)] 
  en <- en[,!apply(assay(en), 2, anyNA)] 
  print("Dimensions of tumor and adj nml dataframes (duplicates removed + pts with NA values removed):")
  print(dim(et)); print(dim(en))
  
  #Filter out genes where <20% of patients have a non zero expression value
  et <- et[rowSums(assay(et)==0)<=ncol(et)*.80,] #select genes w/ less than 80% zeros
  en <- en[rowSums(assay(en)==0)<=ncol(en)*.80,] #select genes w/ less than 80% zeros
  print("Dimensions of tumor and adj nml dataframes (duplicates removed + pts with NA values removed + 20% filter):")
  print(dim(et)); print(dim(en))
  
  #get the genes in common between the two
  genes_in_common <- intersect(rownames(et), rownames(en))
  et <- et[genes_in_common,]
  en <- en[genes_in_common,]
  print("Dimensions of tumor and adj nml dataframes (duplicates removed + pts with NA values removed + 20% filter + intersection):")
  print(dim(et)); print(dim(en))
  
  return(list(et=et, en=en))
}


centroid_plot_elem <- function(){
  list(ggplot2::theme_classic(), 
         geom_point(size=3),
         geom_text(aes(label=Type)))
}

returnDeMirnaLimma <- function(expr, design, colOfInt, pval=1, logFC=0, retAll=F){
  fit_t <- lmFit(object = expr, design = design)
  ebfit_t <- eBayes(fit_t)
  if(retAll == T){
    topTableRes_t <- topTable(ebfit_t, coef = colOfInt, number = Inf) 
    return(topTableRes_t)
  }
  
  topTableRes_t <- topTable(ebfit_t, coef = colOfInt, number = nrow(assay(expr)), adjust.method = "BH", p.value = pval, lfc = logFC) 
  #topTableRes_t2 <-topTableRes_t[{topTableRes_t$logFC > logFC} | {topTableRes_t$logFC < logFC},] 
  return(topTableRes_t)
}

assay_to_df <- function(sum_expt, tum_type, gene){
  data.frame(assay(sum_expt[gene,])) %>% 
    t %>% 
    as_tibble() %>%
    mutate(tum_type=tum_type)
}   


returnClassif <- function(tum_matr, tum_res, gene){
  res <- {assay(tum_matr[gene,]) > {tum_res %>% filter(geneNm == gene) %>% pull(lowerBound)}} %>%
    ifelse(1,0) %>% 
    as.data.frame() %>% 
    t() %>% 
    as.data.frame() %>% rename(!!paste0("classif_", gene) := gene)
  return(res)
}

calc_days_last_fu <- function(days_to_death, days_to_last_follow_up){
  days_last_fu <- ifelse(is.na(days_to_death), days_to_last_follow_up, days_to_death)
  days_last_fu <- ifelse(days_last_fu < 1, 1, days_last_fu)
  days_last_fu <- ifelse(days_last_fu > 365.25 * 5, 365.25 * 5, days_last_fu)
  days_last_fu <- as.data.frame(days_last_fu)
  return(days_last_fu)
}

calc_vital_stat <- function(vital_stat, days_last_fu ){
  vital_stat <- ifelse(vital_stat == "alive", 0, 1)
  vital_stat <- ifelse(days_last_fu >= 365.25 * 5, 0, vital_stat)
  vital_stat <- as.data.frame(vital_stat)
  return(vital_stat)
}
```

---

Analysis by Daniel Piqué

*daniel.pique@med.einstein.yu.edu*
